# Improper Background Treatment Underestimates Thermometric Performance of Rare Earth Vanadate and Phosphovanadate Nanocrystals

**DOI:** 10.1021/acsomega.4c04835

**Published:** 2024-08-01

**Authors:** Rafael Vieira Perrella, Gustavo Derroso, Paulo Cesar de Sousa Filho

**Affiliations:** Department of Inorganic Chemistry, Institute of Chemistry, Universidade Estadual de Campinas (Unicamp), R. Monteiro Lobato, 270, Campinas, São Paulo 13083-970, Brazil

## Abstract

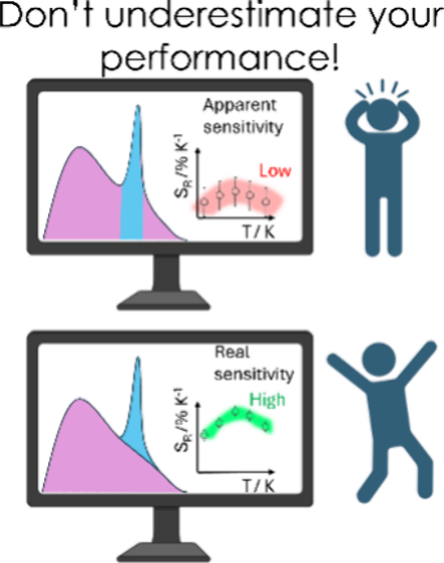

Luminescence thermometry is the state-of-the-art technique for remote nanoscale temperature sensing, offering numerous promising cutting-edge applications. Advancing nanothermometry further requires rational design of phosphors and well-defined, comprehensive mathematical treatment of spectral information. However, important questions regarding improper signal processing in ratiometric luminescence thermometry are continuously overlooked in the literature. Here, we demonstrate that systematic errors arising from background/signal superposition impact the calculated thermometric quality parameters of ratiometric thermometers. We designed ultraviolet-excitable (Y,Eu)VO_4_ and (Y,Eu)(P,V)O_4_ nanocrystals showing overlapped VO_4_^3–^ and Eu^3+^ emissions to discuss systematically how uncorrected background emissions cause magnified (∼10×) temperature uncertainties and undervalued (∼60%) relative thermal sensitivities. Adequate separation of spectral contributions from the VO_4_^3–^ background and the Eu^3+^ signals via baseline correction is necessary to prevent underestimation of the thermometric performances. The described approach can be potentially extended to other luminescent thermometers to account for signal superposition, thus enabling to circumvent computation of apparent, miscalculated thermometric parameters.

## Introduction

Remote temperature probes based on temperature-sensitive luminescence are highly versatile tools for nanomedicine, catalysis, and electronics.^1−4^ Typical nanothermometers used in this context explore the abundant electronic level pattern of trivalent lanthanoid ions (Ln^3+^), which enable tailoring absorption and emission from the ultraviolet (UV) to the near-infrared (NIR) by the adequate choice of activators and sensitizers.^5,6^ Furthermore, the narrow line width and minimal spectral overlap between 4f–4f transitions also prevents systematic errors, which provides enhanced accuracy for the thermometric determinations. While several optical parameters arising from Ln^3+^ luminescence are often available for thermal correlations,^7,8^ the luminescence intensity ratio (LIR) between two electronic transitions (or Stark components of a single excited level) in thermal equilibrium is by far the preferred choice for calculating absolute temperatures in both primary and nonprimary optical thermometers.^9−12^ This method is not only easily implemented but it is also considered robust against experimental or sample-related conditions, such as fluctuations in excitation intensity, sample geometry, or concentration of luminescent probes.^13^

Although ratiometric thermal correlations become progressively widespread, recent investigations have raised concerns about the precision and accuracy of temperature determination using LIR thermometry. Following the landmark work of Labrador-Páez et al.,^14^ several studies have demonstrated the impact of various environmental and experimental factors on the luminescence spectra and emission decays of Ln^3+^-based luminescent materials.^9,10,15^ These factors include low signal-to-noise (SNR) ratios and the presence of artifacts introducing biases in the thermometric correlations. For instance, SNR quantifies the readout intensity toward the random intensity fluctuations during the measurement, which depends on excitation exposure times, brightness of the nanoprobe, and extinction of emission photons in an opaque medium.^10,13,14^ In addition, reliability (i.e., difference between measured and real temperature) is affected by a distorted luminescence spectral distribution arising from wavelength-dependent transmission by the sample, wavelength-dependent self-absorption by the thermometer, or modified density of optical states around the optical probe.^9,14−17^ Finally, the presence of intruding transitions within analyzed spectral ranges in LIR introduces misinterpretation, as described in cases involving Er^3+^- or Nd^3+^-based thermometry.^14,18,19^ Such effects are not inherent properties of the nanothermometers, and the random nature of some of them makes it challenging to adequately process the acquired emission spectra, hindering unequivocal thermal readouts.

Here, we show that lack of background emission treatment is also an origin of inaccuracy, leading to incorrect values of relative thermal sensitivities and temperature uncertainties. Despite the high number of works dealing with potentially superposed signals for luminescence thermometry, only scarce works have investigated the artifacts arising from background emissions to date. We investigated this issue by elaborating Eu^3+^-doped yttrium vanadate (YVO_4_) and yttrium phosphovanadate (Y(V,P)O_4_) particles, which are UV-excited phosphors showing both narrow and broad bands arising from Eu^3+^ and VO_4_^3–^ emissions. The intrinsic spectral overlap between ^3^T_1,2_ → ^1^A_1_ (VO_4_^3–^) and ^5^D_0_ → ^7^F_J_ (Eu^3+^) transitions is a fruitful example to demonstrate the general effect of background emissions in the sensor performance. The idea is not only providing insights to decide whether the temperature correlations are reliable or not but also presenting strategies to circumvent this realistic practical artifact.

## Methods

Eu^3+^-doped yttrium vanadate and phosphovanadate nanoparticles were synthesized using a colloidal coprecipitation reaction under hydrothermal conditions, both with and without citrate groups as capping agents. For the preparation of vanadate or phosphovanadate nanoparticles without citrates as stabilizers, the process involved heating a mixture of aqueous rare earth chlorides (YCl_3_ and EuCl_3_, 99.9%Y^3+^, 0.1%Eu^3+^, mol/mol) and a combination of aqueous ammonium metavanadate (NH_4_VO_3_) and ammonium hydrogen phosphate ((NH_4_)_2_HPO_4_) in the desired V/P molar ratios. The reactions were performed at 180 °C for 20 h at pH 3. In contrast, for the synthesis of citrate-stabilized vanadate/phosphovanadate nanoparticles, the processes began with mixing rare earth chlorides (99.9%Y^3+^, 0.1%Eu^3+^, mol/mol) and sodium citrate (Na_3_cit·2H_2_O) in water, followed by the addition of an aqueous solution containing sodium orthovanadate (Na_3_VO_4_) and ammonium hydrogen phosphate ((NH_4_)_2_HPO_4_) in the desired V/P molar ratios. This synthesis was carried out at 200 °C for 24 h. A detailed description of the experimental procedures, characterization techniques, and data processing is provided in the Supporting Information.

## Results and Discussion

**Figure 1:**
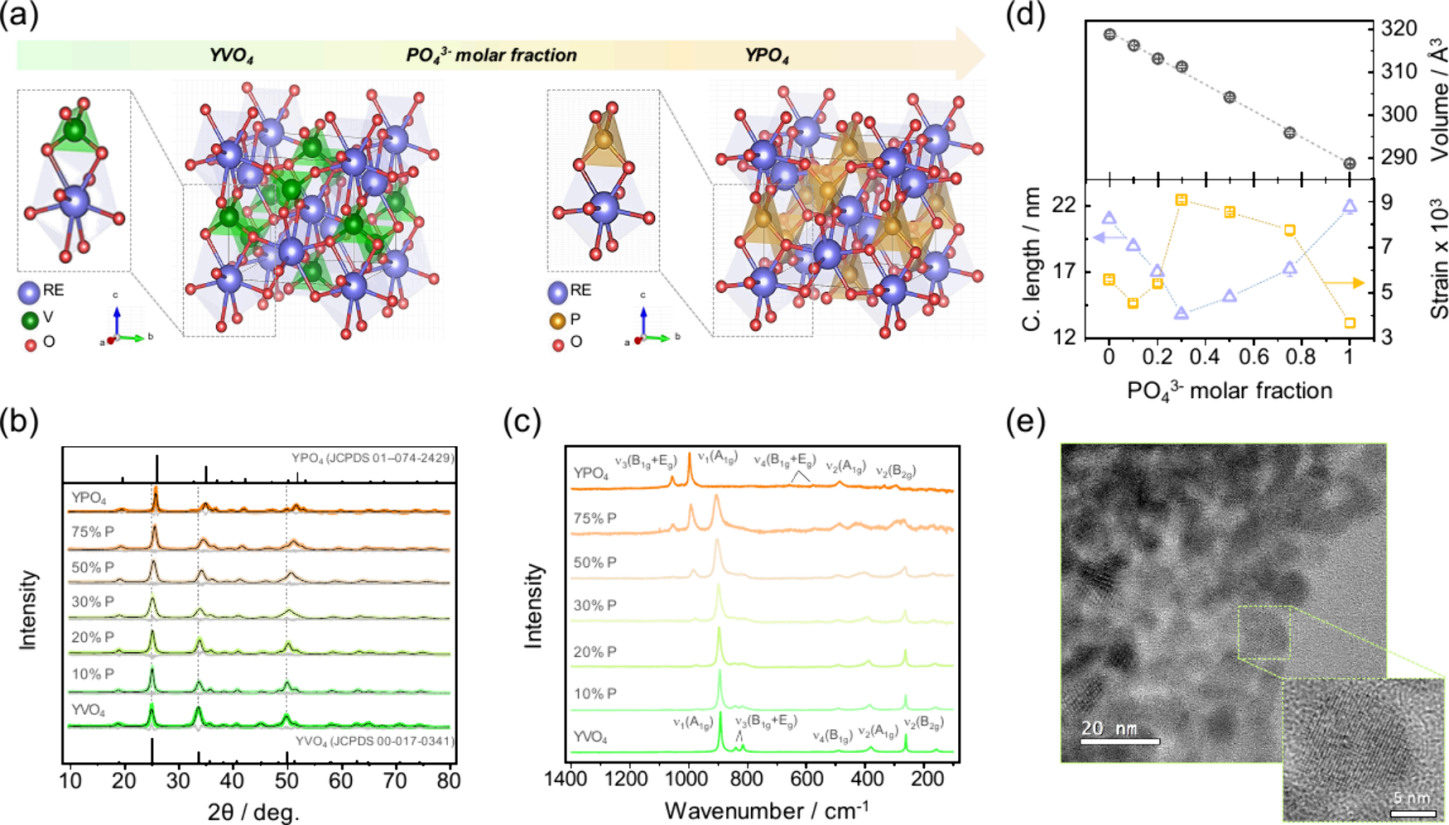
Structural properties of the (Y_0.999_Eu_0.001_)(V_1–*x*_P_*x*_)O_4_ nanocrystals. (a) Representation of the unit cell and coordination polyhedra in the xenotime-type structure (*I*4_1_/*amd* space group) of YVO_4_ and YPO_4_. (b) Powder X-ray diffraction patterns and (c) Raman spectra of (Y_0.999_Eu_0.001_)(V_1–*x*_P_*x*_)O_4_ nanocrystals. (d) Evolution of unit cell volumes, coherence lengths (c. length), and microstrain with respect to PO_4_^3–^ molar ratio. (e) Representative TEM image of (Y_0.999_Eu_0.001_)(V_0.8_P_0.2_)O_4_ particles showing the internal structure of a single particle (inset).

Anhydrous YVO_4_ and YPO_4_ crystallize in the same tetragonal xenotime-type structure (Figure 1a) forming solid solutions at the complete range of compositions.^20^ Powder X-ray diffraction (XRD) patterns and Raman spectra evidenced the formation of single-phase tetragonal solids (*I*4_1_/*amd* space group) and the homogeneous incorporation of PO_4_^3–^ into the YVO_4_ lattice (Figure 1b,c). This was further corroborated by the linear constriction of unit cell volumes of Y(V_1–*x*_P_*x*_)O_4_:Eu^3+^ particles upon higher PO_4_^3–^ molar fractions (Figure 1d), which was also confirmed by Rietveld refinement of experimental XRD data (Table S1 and Figure S1). Raman and infrared (FTIR) spectra attested the occupancy of tetrahedral sites distorted to a *D*_2d_ symmetry by VO_4_^3–^/PO_4_^3–^ species, in accordance with the *I*4_1_/*amd* structure (Figure 1c and Figure S2). The microstructural alterations caused by PO_4_^3–^ groups included a preferential growth in the (200) plane (Figure 1b) as well as lower crystalline coherence lengths and higher microstrains (Figure 1d). By contrast, additional inclusion of PO_4_^3–^ ions (i.e., *x* > 0.3, with *x* = PO_4_^3–^/(PO_4_^3–^ + VO_4_^3–^) mol/mol) led to larger crystalline domains and decreased lattice defects. These results highlight the chemical homogeneity of the particles and the effective control of structural properties through the employed colloidal synthesis.

Transmission electron microscopy (TEM) images of a representative phosphovanadate sample revealed weakly agglomerated nanocrystals with sizes of 15–40 nm (Figure 1e and Figure S3), in agreement with the dynamic light scattering (DLS) results (Figure S4). The presence of lattice fringes throughout the particle volume indicates a high crystalline quality, which is crucial for a high luminescence output.^21^ Thanks to the surface stabilization provided by citrate groups [FTIR, υ_as_(COO^–^) = 1560 cm^–1^, υ_s_(COO^–^) = 1437 cm^–1^, υ(CH) = 2853 + 2921 cm^–1^, and υ(OH) = 3315 cm^–1^, Figure S5], similar DLS-particle size distributions were achieved regardless of sample composition (Figure S4). As a matter of comparison, a hydrothermal protocol without sodium citrate resulted in Y(V_1–*x*_P_*x*_)O_4_:Eu^3+^ nanoparticles with variable sizes (518 ± 11 nm to 63 ± 3 nm) with increasing PO_4_^3–^ fractions (Figure S6). We therefore conclude that citrate groups efficiently provide surface stabilization and regulate growth rates during precipitation, thus yielding highly crystalline nanoparticles for UV-excited luminescent nanothermometry.

Aiming to develop a ratiometric luminescent thermometer based on VO_4_^3–^ and Eu^3+^ emissions, a low Eu^3+^ doping ratio (*x* = 0.1% mol/mol with respect to Y^3+^) was selected. This enabled to achieve Eu^3+^ and VO_4_^3–^ with similar absolute intensities for a Y_0.999_Eu_0.001_VO_4_ sample at 77 K (Figure S7). Considering the high thermal quenching of vanadate emissions,^22,23^ we also prepared samples containing varying amounts of PO_4_^3–^ in the YVO_4_ host. The partial dilution reduces the VO_4_^3–^ → VO_4_^3–^ energy transfer probability, thus enhancing the ^3^T_1,2_ → ^1^A_1_ emissions (Figure S7). In turn, similar Eu^3+^ and VO_4_^3–^ emission intensities were produced under 280 nm excitation even at 297 K. Using as criteria the better crystalline quality (i.e., higher coherence length and low strain, Figure 1d) combined to higher VO_4_^3–^ emission intensities at 297 K and better signal-to-noise ratio (Figure S7), we selected a 20% mol/mol PO_4_^3–^ concentration (i.e., Y_0.999_Eu_0.001_(V_0.8_P_0.2_)O_4_) for thermometric studies in comparison to Y_0.999_Eu_0.001_VO_4_.

**Figure 2:**
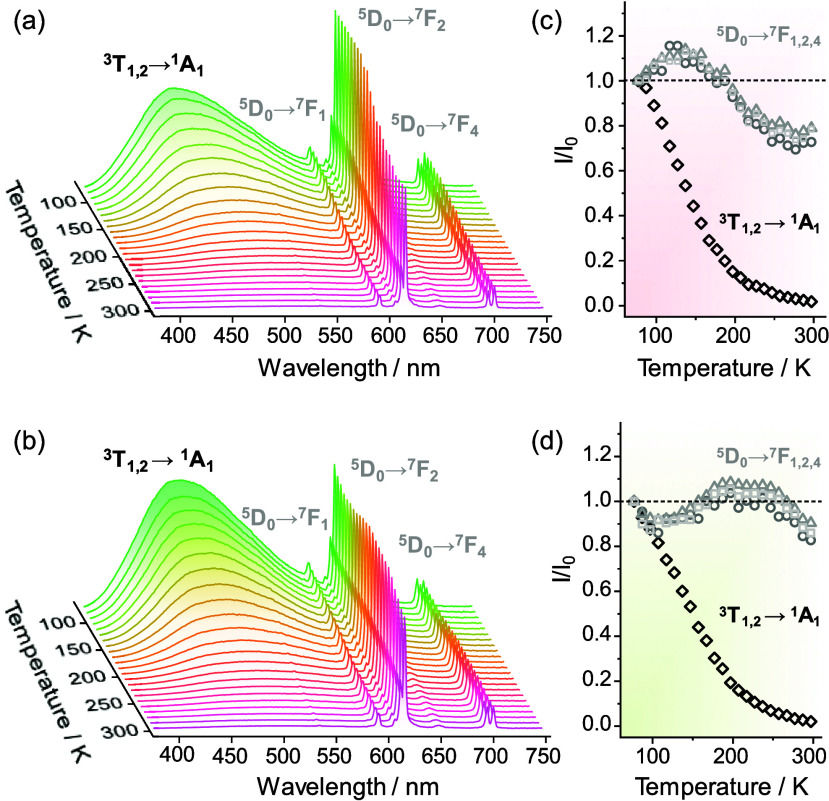
Temperature-dependent emission spectra (λ_exc._= 280 nm, 77–297 K) of the (a) Y_0.999_Eu_0.001_VO_4_ and (b) Y_0.999_Eu_0.001_(V_0.8_P_0.2_)O_4_ nanocrystals. (c, d) Integrated intensities of the VO_4_^3–^ (^3^T_1,2_ → ^1^A_1_, black diamonds) and Eu^3+^ [^5^D_0_ → ^7^F_1,2,4_, gray circles (*J* = 1), triangles (*J* = 2), and squares (*J* = 4)] transitions normalized to their corresponding values at 77 K (*I*_0_).

Temperature-dependent luminescence spectra (Figure 2a,b) unveiled only partially altered intensities of the ^5^D_0_ → ^7^F_1–4_ Eu^3+^ transitions in the 77–297 K range, in contrast to the significant thermal quenching of the ^3^T_1,2_ → ^1^A_1_ VO_4_^3–^ emissions (Figure 2c,d). Such an inhomogeneous behavior arises from dissimilar thermal dependences of Eu^3+^ and VO_4_^3–^ emitting states, which provides an attractive pathway for temperature determination, as extensively pointed out by us^11^ and by many authors.^22−24^ However, the robustness of this approach depends critically on the absence of signal superposition and intruding emissions in the analyzed emission ranges. This is because such signals often result in systematic artifacts in ratiometric luminescence thermometry, where thermometric performances and reported readouts become erroneous.^10,14,15,18^

**Figure 3:**
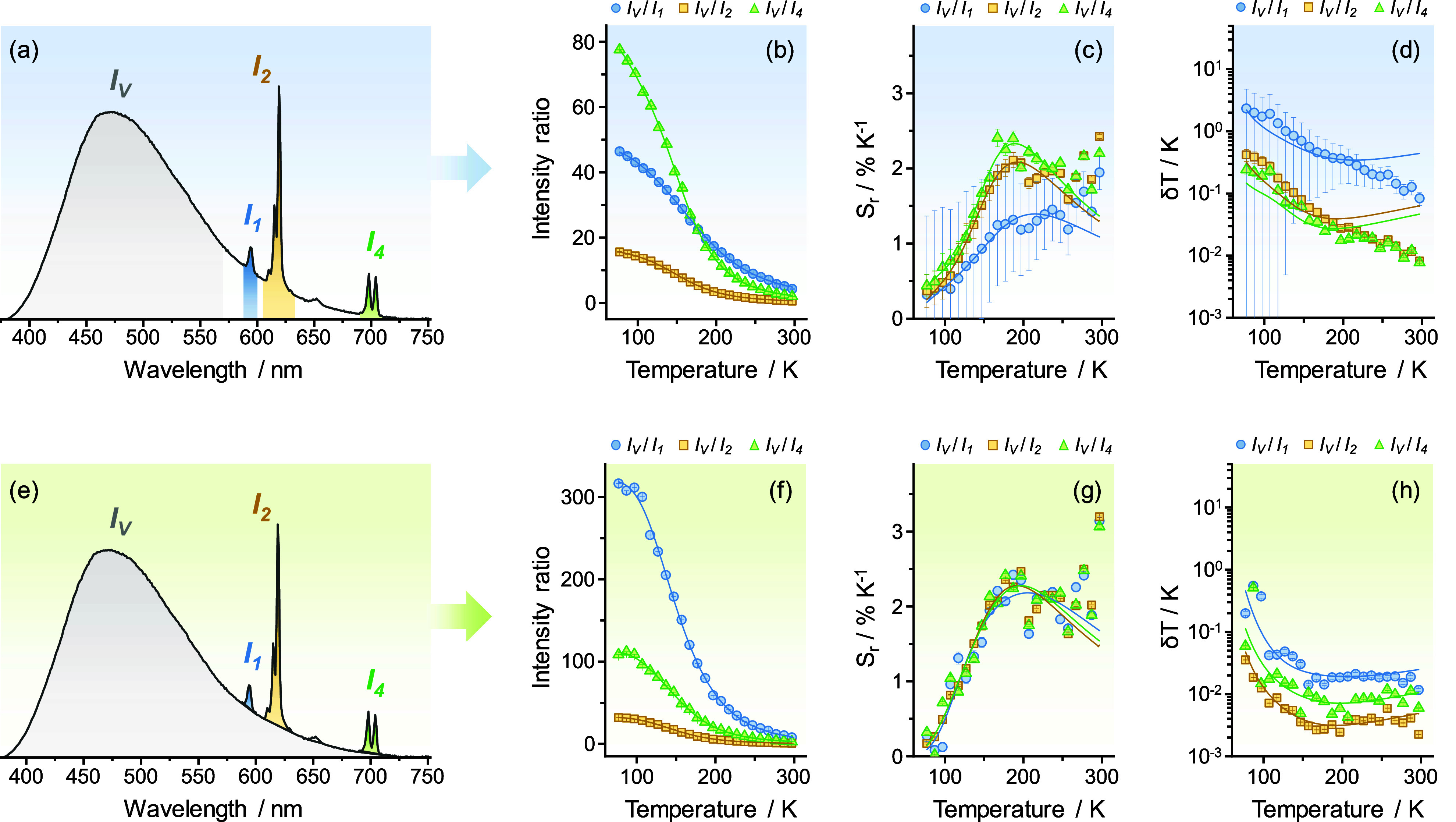
Impact of background emission on the thermometric performance of Y_0.999_Eu_0.001_(V_0.8_P_0.2_)O_4_ nanocrystals. (a, e) Emission spectra (λ_exc_ = 280 nm, 77 K) illustrating the two spectral processing approaches to derive the intensity ratios. *I*_V_ denotes the integrated intensity of the ^3^T_1,2_ → ^1^A_1_ VO_4_^3–^ transitions, whereas *I*_1_, *I*_2_, and *I*_4_ correspond to the integrated intensities of the ^5^D_0_ → ^7^F_1,2,4_ Eu^3+^ transitions, respectively. Intensity ratios were calculated (a–d) neglecting signal superposition and (e–h) including a correction of the VO_4_^3–^ emission background to compute the intensities of the Eu^3+^ signals. Temperature dependence of the (b, f) intensity ratios, (c, g) relative thermal sensitivities (*S*_r_), and (d, h) temperature uncertainties (δ*T*). Solid lines in panels (b, f) represent the best fits using eq 1 (*r*^2^ > 0.998), while solid lines in panels (c, d, g, h) correspond to the mathematical derivation to model *S*_r_ and δ*T*. Fitting parameters are summarized in Table S2.

The impact of background signals on the thermometry quality parameters is evidenced by comparing the luminescence of the Y_0.999_Eu_0.001_(V_0.8_P_0.2_)O_4_ and Y_0.999_Eu_0.001_VO_4_ samples (Figure 3 and Figure S8), where the phosphovanadate solid shows an even higher spectral overlap between VO_4_^3–^ and Eu^3+^ emissions than the unmixed vanadate sample. We evaluated two approaches to assess thermometric parameters, the first one neglecting signal superposition, and the second one including a correction of the VO_4_^3–^ emission background to compute the intensities of the Eu^3+^ signals. The thermometric parameters (Δ) were defined as the integrated intensity ratio between the VO_4_^3–^ emission (*I*_V_) and each of the Eu^3+^ transitions arising from the ^5^D_0_ excited state, namely ^5^D_0_ → ^7^F_1_ (*I*_1_), ^5^D_0_ → ^7^F_2_ (*I*_2_), and ^5^D_0_ → ^7^F_4_ (*I*_4_). Given the broad spectral width of the VO_4_^3–^ emission band, spectral deconvolution of the broadband emissions was carried out considering Intensity vs wavenumbers rather than Intensity vs wavelength (Figure S9). To ensure the conservation of energy remains valid, we applied the Jacobian transformation^25^ to all spectra. In the first analyzed approach, the vanadate signal was computed in the 380–570 nm range (17544–26316 cm^–1^) while the Eu^3+^ emissions (^5^D_0_ → ^7^F_1_: 16722–16978 cm^–1^, ^5^D_0_ → ^7^F_2_: 15949–16502 cm^–1^, and ^5^D_0_ → ^7^F_4_: 14084–14451 cm^–1^) were integrated without background treatment (Figure 3a). The temperature dependence of the intensity ratios displayed sigmoidal profiles and were modeled in terms of the Mott-Seitz equation,^26,27^ considering two nonradiative recombination channels associated with the ^3^T_1,2_ → ^1^A_1_ VO_4_^3–^ transitions (eq 1):
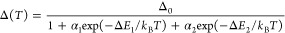
1where Δ_0_ is the Δ parameter when *T* → 0 K, α_1_ and α_2_ are the ratio between the nonradiative and radiative probabilities of the deactivation channels for the ^3^T_1_ → ^1^A_1_ and ^3^T_2_ → ^1^A_1_ transitions, and Δ*E*_1_ and Δ*E*_2_ denote the activation energies for the thermal quenching of the corresponding excited states. The use of eq 1 is necessary because assuming a single thermal quenching pathway resulted in inadequate modeling of experimental points at temperatures exceeding 225 K (Figure S10). The final Δ vs temperature calibration curves showed good correlation coefficients with experimental data (*r*^2^ > 0.998) regardless of the choice of the ^5^D_0_ → ^7^F_J_ (*J* = 1, 2 or 4) Eu^3+^ emissions (Figure 3b and Table S2).

The spectral superposition of the VO_4_^3–^ band is higher for the ^5^D_0_ → ^7^F_1_ (594 nm) emission than for the ^5^D_0_ → ^7^F_2_ (619 nm) and ^5^D_0_ → ^7^F_4_ (698 nm) Eu^3+^ emissions. Consequently, the Δ = *I*_V_/*I*_1_ parameter becomes underestimated because *I*_1_ has a large contribution arising from the VO_4_^3–^ emission if integration is performed without correction. This effect was less pronounced for *I*_V_/*I*_2_ and *I*_V_/*I*_4_ ratios due to the high intensity of the ^5^D_0_ → ^7^F_2_ transition (*I*_2_) and the lower vanadate emission intensity above 680 nm, respectively.

To quantify the thermometric performance, the relative thermal sensitivity (*S*_r_) was calculated as a function of temperature as follows^28^:
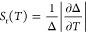
2

As expected, *S*_r_ presented bell-shaped profiles peaking around 192–214 K (Figure 3c). Because the different Eu^3+^ emissions used for the thermometric correlations arise from the same emitting state (^5^D_0_), they should ideally yield similar thermal sensitivities after combination with the integrated VO_4_^3–^ intensities to compute the Δ parameters. Nonetheless, maximum relative sensitivities (*S*_m_) were not similar among analyzed ratios and decreased progressively from *I*_V_/*I*_4_ (*S*_m_ = 2.33 ± 0.10% K^–1^) to *I*_V_/*I*_2_ (*S*_m_ = 2.08 ± 0.10% K^–1^), and *I*_V_/*I*_1_ (*S*_m_ = 1.39 ± 0.52% K^–1^). This trend is due to the increasing contribution of the VO_4_^3–^ band to the integration limits of the Eu^3+^ signals, also emphasizing how background emissions may cause misleading sensitivity values in luminescence thermometry. The spectral overlap with a non-negligible broadband background introduces an additive temperature-dependent term on the Eu^3+^ integrated intensities, which results in an apparent, underestimated thermometric parameter Δ. This ultimately causes a reduction in the  term of eq 2 if this superposition is not corrected, thus negatively affecting the relative thermal sensitivity values. The general effect of a spectral overlap of the background emissions is discussed mathematically in the Supporting Information (eqs S1–S11). Our conclusions also align with the discussion proposed by Brites et al.^28^ upon band overlap on previously reported Pr^3+^/Yb^3+^/Tm^3+^-doped NaYF_4_ nanocrystals.^29^

The effects of uncorrected background emissions causing deviations in the thermometric parameters also include lower apparent signal-to-noise ratios (i.e., ratio between peak and baseline intensities), consequently causing a less precise readout. This was evaluated by determining the minimum expected statistical temperature uncertainty (δ*T*) using eq 3(10):
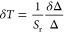
3where δΔ/Δ stands for the relative uncertainty in the determination of Δ, which has an inverse dependence on the signal-to-noise ratio (SNR) of each transition.^10^ For Eu^3+^ transitions (*I*_1_, *I*_2_ and *I*_4_), SNR raised exponentially with temperature due to the reduced contribution of VO_4_^3–^ emission in the monitored spectral ranges. This correlates to a lower δΔ/Δ and a decreased δ*T* as a function of temperature (Figure 3d and Figure S11). As expected, the *I*_V_/*I*_1_ ratio displayed the highest δ*T* values, ranging from 2.33 ± 2.44 K to 0.08 ± 0.02 K between 77 e 297 K. These temperature uncertainties are 1 order of magnitude higher than those observed for *I*_V_/*I*_2_ and *I*_V_/*I*_4_ ratios (Figure 3d).

To overcome these limitations, we applied a spectral separation of VO_4_^3–^ luminescence from the Eu^3+^ emissions (Figure 3e) to calculate thermometric parameters. First, the baselines of spectral regions corresponding to the ^5^D_0_ → ^7^F_J_ Eu^3+^ transitions were corrected to eliminate the vanadate contribution from the background. Then, the VO_4_^3–^ emission envelope within 380–750 nm range was determined by linear interpolation after removal of Eu^3+^ signals. As a result, the intensity ratios exhibited the expected tendency for Δ_0_ at 77 K: *I*_V_/*I*_1_ > *I*_V_/*I*_4_ > *I*_V_/*I*_2_ (Figure 3f), which is consistent with the relative intensities of VO_4_^3–^ and Eu^3+^ transitions. The *S*_r_ values showed lower relative errors (Figure 3g), while *S*_m_ values around 194–204 K were nearly the same for the three intensity ratios (*S*_m_ = 2.18 ± 0.06% K^–1^, *S*_m_ = 2.27 ± 0.01% K^–1^, and *S*_m_ = 2.27 ± 0.02% K^–1^ for *I*_V_/*I*_1_, *I*_V_/*I*_2_, and *I*_V_/*I*_4_, respectively). The background treatment resulted in an improved SNR for the ^5^D_0_ → ^7^F_J_ Eu^3+^ transitions and consequently a reduced δΔ/Δ and δ*T* values (Figure 3h and Figure S11). Experimental temperature uncertainties presented lower dispersions being better adjusted to the proposed model. In addition, *I*_V_/*I*_2_ ratio yielded δ*T* ranging from 0.55 ± 0.04 K to 0.011 ± 0.001 K across the 77–297 K range. This represents a remarkable 10-fold reduction in temperature uncertainties compared to the previous analysis. Ultimately, the δ*T* decreased with the increasing of the SNR involving both VO_4_^3–^ and Eu^3+^ emissions, with the *I*_V_/*I*_2_ ratio producing the lowest values (Figure 3h and Figure S11). This supports the observation that ^5^D_0_ → ^7^F_2_ transition produces the most intense Eu^3+^ emission (Figure 2a,b). This outcome agrees with the recent analyses conducted by van Swieten et al.^15^ and Brites et al.,^10^ emphasizing that higher SNR leads to a more precise temperature assessment. Similar trends were observed for Y_0.999_Eu_0.001_VO_4_ nanocrystals (Figure S8).

An alternative approach to deal with the background artifacts involves computing a fraction of the VO_4_^3–^ emission band (excluding overlap with the ^5^D_0_ → ^7^F_1_ Eu^3+^ transition) (Figure S12). This method employs narrower VO_4_^3–^ integration boundaries, potentially inducing higher temperature uncertainties due to decreased SNR.^15^ Despite this expectation, similar *S*_r_ and δ*T* results were achieved when compared to analyzing the entire VO_4_^3–^ band. This is also a consequence of the broad spectral width of the emission, which minimizes detrimental effects on thermometric performance resulting from different integration limits. Consequently, addressing reliability issues related to background emissions is primarily attainable through baseline corrections of the considered electronic transitions rather than selecting specific integration regions.

**Figure 4:**
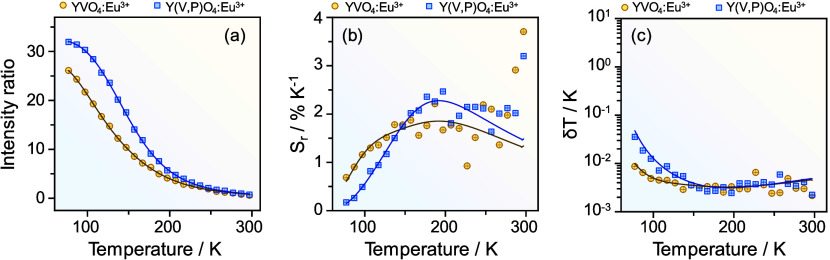
Comparative thermometric performances of the Y_0.999_Eu_0.001_VO_4_ and Y_0.999_Eu_0.001_(V_0.8_P_0.2_)O_4_ nanocrystals in terms of (a) integrated intensity ratio (*I*_V_/*I*_2_), (b) relative thermal sensitivity (*S*_r_), and (c) temperature uncertainty (δ*T*). Solid lines in panel (a) represent the best fits using eq 1 (*r*^2^ > 0.998), while solid lines in panels (b, c) correspond to the mathematical derivation to model *S*_r_ and δ*T*. Fitting parameters are summarized in Table S2.

The above-described discussion provided solid basement for comparatively evaluating the thermal performances of the Y_0.999_Eu_0.001_VO_4_ and Y_0.999_Eu_0.001_(V_0.8_P_0.2_)O_4_ nanocrystals. This investigation focused on the *I*_V_/*I*_2_ ratio, as it offered superior thermometric correlations (Figure 3). The temperature calibration curves (Figure 4a) yielded the following activation energies (eq 1) for thermal quenching of the ^3^T_1,2_ → ^1^A_1_ VO_4_^3–^ transitions: Δ*E*_1_ = 978 ± 92 cm^–1^ and Δ*E*_2_ = 328 ± 23 cm^–1^ for Y_0.999_Eu_0.001_VO_4_, and Δ*E*_1_ = 1019 ± 88 cm^–1^ and Δ*E*_2_ = 438 ± 44 cm^–1^ for Y_0.999_Eu_0.001_(V_0.8_P_0.2_)O_4_ (Table S2). These nonradiative recombination channels (Δ*E*_1_ and Δ*E*_2_) represent the energy gap between the bottom of the potential energy curve of the ^3^T_2_ and ^3^T_1_ emitting states and the crossover point with the VO_4_^3–^ ground state (^1^A_1_) or the Eu^3+^ excited states.^11^ The results confirm that the barrier for thermal deactivation of the ^3^T_1,2_ → ^1^A_1_ transitions is slightly higher when VO_4_^3–^ centers are diluted in the YVO_4_ lattice by the presence of PO_4_^3–^ groups. This is because homogeneous incorporation of PO_4_^3–^ in the crystalline lattice decreases the energy propagation rate through VO_4_^3–^ groups and enhances radiative decay intensities.^30,31^ As a result, the emission becomes less prone to nonradiative decays upon heating (Figures 2a,b and 4a).

Because relative thermal sensitivities are proportional to Δ*E*, a higher maximum sensitivity is expected for the Y_0.999_Eu_0.001_(V_0.8_P_0.2_)O_4_ nanoparticles. Indeed, the phosphovanadate nanocrystals exhibited a *S*_m_ of 2.27 ± 0.01% K^–1^ at 191 K, surpassing the 1.85 ± 0.01% K^–1^ observed for vanadate nanocrystals (Figure 4b). Conversely, Y_0.999_Eu_0.001_VO_4_ nanoparticles displayed more sensitive correlations at temperatures below 140 K due to the maximum variation of the Δ parameter occurring at lower temperatures (Figure S13). At temperatures exceeding 277 K, the low VO_4_^3–^ emission intensities make the Δ parameter to approach to zero, and consequently, *S*_r_ to infinity. This is particularly pronounced for Y_0.999_Eu_0.001_VO_4_ solids and obviously lacks physical significance.^7,32^ Operational ranges for thermometry were therefore established based on two conditions: (i) *S*_r_ > 1% K^–1^ and (ii) VO_4_^3–^ emission intensity exceeding 3% of its integrated intensity at 77 K. The first condition is widely accepted for practical purposes^11,33^ while the second one ensures reliability in the thermal performance. Accordingly, operational ranges for Y_0.999_Eu_0.001_VO_4_ and Y_0.999_Eu_0.001_(V_0.8_P_0.2_)O_4_ nanocrystals were determined as 97 to 277 K and 127 to 287 K, respectively. These ranges align perfectly with temperature requirements in superconducting magnets, aerospace, and macromolecular crystallography,^34−36^ showcasing potentiality of this system for sensitive and accurate temperature evaluation. Indeed, temperature uncertainties ranged between (0.35 ± 0.01) × 10^–1^ K and (0.02 ± 0.01) × 10^–1^ K (Figure 4c) for both compositions. The reduced δ*T* values for Y_0.999_Eu_0.001_VO_4_ nanocrystals below 140 K stems from higher thermal sensitivity in this range instead of lower SNR (Figure 4b and Figure S13). A comparative analysis highlights the favorably thermometric capabilities of both Y_0.999_Eu_0.001_VO_4_ and Y_0.999_Eu_0.001_(V_0.8_P_0.2_)O_4_ samples compared to other luminescent nanothermometers, whether single- or dual-center emitting (Table S3). Hence, our results suggest that prepared samples emerge as promising candidates for luminescent nanothermometry applications, specially within the cryogenic temperature range.

## Conclusions

In summary, background emissions are a realistic practical problem on ratiometric optical thermometry. We hereby describe how neglecting this issue negatively affects the thermometric correlations of a dual-center thermometer based on VO_4_^3–^ and Eu^3+^ emissions in Y_0.999_Eu_0.001_VO_4_ and Y_0.999_Eu_0.001_(V_0.8_P_0.2_)O_4_ nanocrystals. Applying appropriate baseline correction ensures reliable relative thermal sensitivities, also significantly reducing the temperature uncertainties. The Y_0.999_Eu_0.001_VO_4_ and Y_0.999_Eu_0.001_(V_0.8_P_0.2_)O_4_ solids exhibited outstanding UV-excited thermometric performance, achieving maximum relative sensitivities and temperature uncertainties around 2% K^–1^ (at 191 K) and 0.03–0.002 K, respectively. The particles also showed a broad *S*_r_ > 1% K^–1^ operational range (97 to 287 K), which is useful for cryogenic applications. Our work highlights that temperature determination depends not only on measurement conditions or sample characteristics but also on spectral artifacts inherent to all luminescence spectra. This insight extends beyond the specific case of vanadate or phosphovanadate particles, and similar spectral treatments is recommended to eliminate the impact of spurious signals on the thermometric performances of luminescent nanothermometers with overlapped emission bands.

## Supporting Information Available

Additional experimental details, X-ray diffractograms and results of data refinement, FTIR spectra, TEM images, DLS particle size distributions, emission spectra, spectral analysis of emission bands, additional temperature-dependent spectra, thermometric performance parameters (*S*_r_, δ*T*, and δΔ/Δ), and analysis of spectral overlap on the thermometry quality parameters (PDF)
