# Supplementary material for: Improper Background Treatment Underestimates Thermometric Performance of Rare Earth Vanadate and Phosphovanadate Nanocrystals
Source: ACS Omega. 2024 Aug 1;9(32):34974–80. doi: 10.1021/acsomega.4c04835 (PMC11325507; doi:10.1021/acsomega.4c04835)
Supplement: Supplementary file 1 — ao4c04835_si_001.pdf [file ao4c04835_si_001.pdf]

SUPPORTING INFORMATION

Improper Background Treatment Underestimates Thermometric Performance of Rare Earth Vanadate and Phosphovanadate Nanocrystals

Rafael V. Perrella,[†] *Gustavo Derroso,*[†] *Paulo Cesar de Sousa Filho*[†,*]

[†]Department of Inorganic Chemistry, Institute of Chemistry, Universidade Estadual de Campinas (Unicamp), R. Monteiro Lobato, 270, 13083-970, Campinas, São Paulo, Brazil

*Corresponding author: pcsfilho@unicamp.br

TOC

	Page
1. EXPERIMENTAL ……………………………………………………………...	S3
2. STRUCTURAL ANALYSIS …………………………………………………	S6
3. THERMOMETRIC PROPERTIES ……………………………………...	S13
4. IMPACT OF SIGNAL SUPERPOSITION ON THE THERMOMETRY QUALITY PARAMETERS …………………………………………………	S24
5. REFERENCES ……………………………………………………….....	S28

1. EXPERIMENTAL

Chemicals

Rare earth oxides (Y_2O_3 and Eu_2O_3, 99.99%), ammonium metavanadate (NH_4VO_3, ≥99.0%), sodium orthovanadate (Na_3VO_4, 99.98%), ammonium phosphate dibasic ($(NH_4)_2HPO_4$, ≥98%) and sodium citrate dihydrate [$Na_3C_6H_5O_7.2H_2O$ ($Na_3cit.2H_2O$), ≥99.0%) were used as received from Sigma-Aldrich. Rare earth chloride stock solutions (YCl_3, 0.1 mol L^{-1}, and $EuCl_3$, 0.01 mol L^{-1}) were prepared by dissolving the previously calcinated oxides in concentrated hydrochloric acid (37 wt%, analytical grade, Synth). After complete solubilization, the pH of these solutions was regulated to ~4 by solvent evaporation.

Hydrothermal synthesis using NH_4VO_3

Rare earth phosphovanadate nanocrystals [$Y_{0.999}Eu_{0.001}V_{1-x}P_xO_4$ (x = 0, 0.05, 0.10, 0.20, 0.30, 0.40, and 1)] were synthesized by adapting the protocol described by Ray *et al.*[1] In a general procedure, we dissolved appropriate amounts of $(NH_4)_2HPO_4$ and NH_4VO_3 in 10 mL of deionized H_2O at 70 ˚C to give the desired P/V molar ratios. The total [VO_3^-] + [HPO_4^{2-}] concentration was kept constant at 0.05 mol L^{-1}. After 40 min of stirring, 4.995 mL of YCl_3 and 0.050 mL of $EuCl_3$ stock solutions were added to the phosphate/vanadate solutions. The suspensions were further stirred for 30 min, and then the pH was adjusted to ~3 with a few drops of a freshly prepared 6 mol L^{-1} NH_3 solution. The resulting mixtures were transferred to a Teflon-lined stainless steel autoclave with 20 mL capacity and heated at 180 ˚C for 20 h. As the autoclave cooled to room temperature naturally, the

precipitate was separated by centrifugation and washed twice with deionized H_2O (~50 mL in each cycle). The final solids were dried at 80 ˚C in air for 12 h.

Hydrothermal synthesis using Na_3VO_4 and $Na_3cit.2H_2O$

A modified protocol of the procedure described by Xu *et al.*[2] was used to prepare rare earth phosphovanadate nanoparticles [$Y_{0.999}Eu_{0.001}V_{1-x}P_xO_4$ (x = 0, 0.10, 0.20, 0.30, 0.50, 0.75, and 1)]. First, 4.995 mL of YCl_3 and 0.050 mL of $EuCl_3$ stock solutions were added to a round bottom flask, followed by 5 mL of 0.5 mol L^{-1} $Na_3cit.2H_2O$ solution. After stirring for 1 h at 25 ˚C, we added 5 mL of the phosphate/vanadate solution ($[VO_4^{3-}] + [HPO_4^{2-}] = 0.1$ mol L^{-1}) previously prepared by dissolving the desired molar amounts of Na_3VO_4 and $(NH_4)_2HPO_4$ in deionized H_2O at 70 ˚C. The final mixtures were additionally stirred for 1 h at 25 ˚C and then transferred to a Teflon-lined stainless steel autoclave with 20 mL capacity. The system was maintained at 200 ˚C for 24 h. After naturally cooling to room temperature, the precipitate was isolated by centrifugation, washed twice with deionized H_2O (~50 mL in each cycle), and dried at 60 °C in air for 12 h.

Characterization

Powder X-ray patterns were acquired on a Shimadzu XRD 7000 diffractometer using Cu-Kα radiation (1,5418 Å) in the 10 – 80˚ 2θ range with a screening rate of 2˚ min^{-1} (0.02˚ step). Lattice parameters, coherence length, microstrain, and phase quantification (based on the V/P molar ratio presented in the samples) were determined by Rietveld refinements using MAUD software.[3] The

morphology of the solids was evaluated by transmission electronic microscopy (TEM) using an FEI TECNAI G2 F20 HRTEM microscope operating at 200 kV. Prior to the observation, samples were prepared by directly depositing 5 μL of diluted particle suspensions (0.5 mmol L^{-1} in PO_4^{3-}/VO_4^{3-} ions) on carbon-coated 200 mesh copper grids. After ~1 min, the fluid excess was carefully removed by placing filter paper onto the edge of the grid, followed by air drying. Dynamic light scattering (DLS) measurements were performed on a Malvern Zetasizer Nano ZS instrument with colloidal aqueous suspensions (0.5 mmol L^{-1} in PO_4^{3-}/VO_4^{3-} ions). Raman spectra were obtained on a Horiba T64000 spectrometer (using a He-Ne laser at 632.8 nm as the radiation source), while infrared (ATR-FTIR) measurements were performed on an Agilent Cary 630 spectrometer on a diamond crystal (4 cm^{-1} resolution). Room temperature luminescence experiments were carried out on a Fluorolog 3 (Horiba FL3-22-iHR320) spectrofluorometer using a Hamamatsu R928P photomultiplier tube as a detector and a 450 W xenon arc lamp as an excitation source. For temperature-dependent emission spectra, powder samples were placed in the Linkam Scientific THMS600 temperature-controlled stage, which was set at temperatures ranging from 77 to 297 K. The excitation and emission signals were collected by optical fibers (Wavelength Electronics LFI-3751) and guided to the sample and detector, respectively. The samples were thermalized for 10 min before each acquisition. All luminescence spectra were corrected via software with respect to lamp intensity, optical response, and detector sensitivity.

2. STRUCTURAL ANALYSIS

Table S1. Tetragonal lattice parameters (*a* and *c*), unit cell volume (V), crystalline coherence length (T), and microstrain (ε) values for the $REV_{1-x}P_xO_4$ ($RE = Y_{0.999}Eu_{0.001}$) nanocrystals prepared by hydrothermal synthesis using Na_3VO_4 and $Na_3cit.2H_2O$. R_{wp}, R_p, and R_{exp} correspond to the quality fitting parameters obtained in the refinements

$RE(V_{1-x}P_x)O_4$	*a* / Å	*c* / Å	V / $Å^3$	T / nm	ε $x10^{-3}$	R_{wp}	R_p	R_{exp}
x=0	7.120±0.003	6.289±0.003	318.8±0.3	21.0±0.3	5.6±0.1	0.206	0.154	0.143
x=0.1	7.103±0.002	6.267±0.002	316.2±0.2	18.9±0.2	4.6±0.2	0.182	0.131	0.144
x=0.2	7.080±0.002	6.247±0.002	313.2±0.2	17.0±0.3	5.4±0.2	0.181	0.144	0.138
x=0.3	7.067±0.003	6.233±0.003	311.3±0.3	13.8±0.2	9.1±0.1	0.149	0.112	0.138
x=0.5	7.019±0.003	6.175±0.003	304.2±0.3	15.1±0.3	8.5±0.1	0.152	0.113	0.13
x=0.75	6.962±0.003	6.105±0.003	295.9±0.3	17.2±0.5	7.8±0.3	0.148	0.110	0.133
x=1	6.918±0.002	6.034±0.002	288.8±0.2	21.9±0.6	3.7±0.2	0.227	0.174	0.124

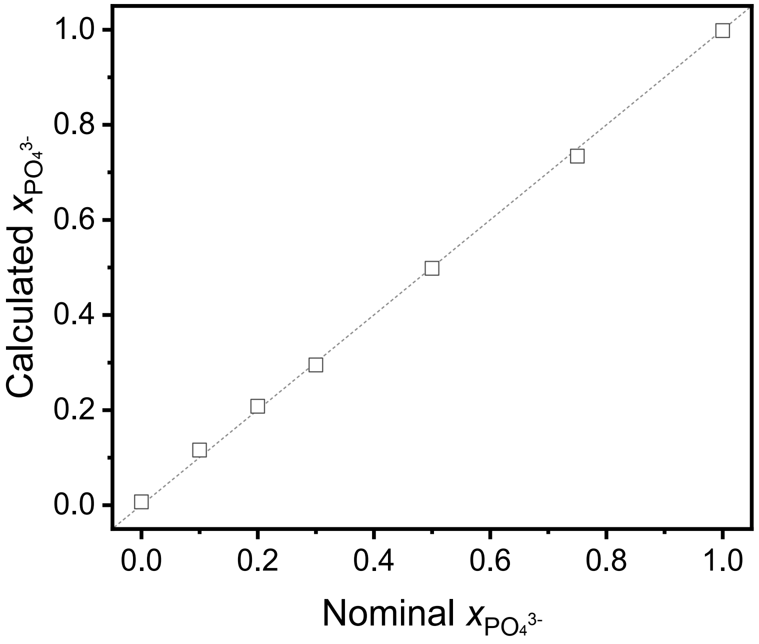

Figure S1. Relationship between nominal and calculated PO_4^{3-} molar fractions for the $Y_{0.999}Eu_{0.001}V_{1-x}P_xO_4$ nanocrystals prepared by hydrothermal synthesis using Na_3VO_4 and $Na_3cit.2H_2O$. Calculated values were obtained from Rietveld refinement of experimental XRD data. Dashed line is a visual guide corresponding to y(x) = x.

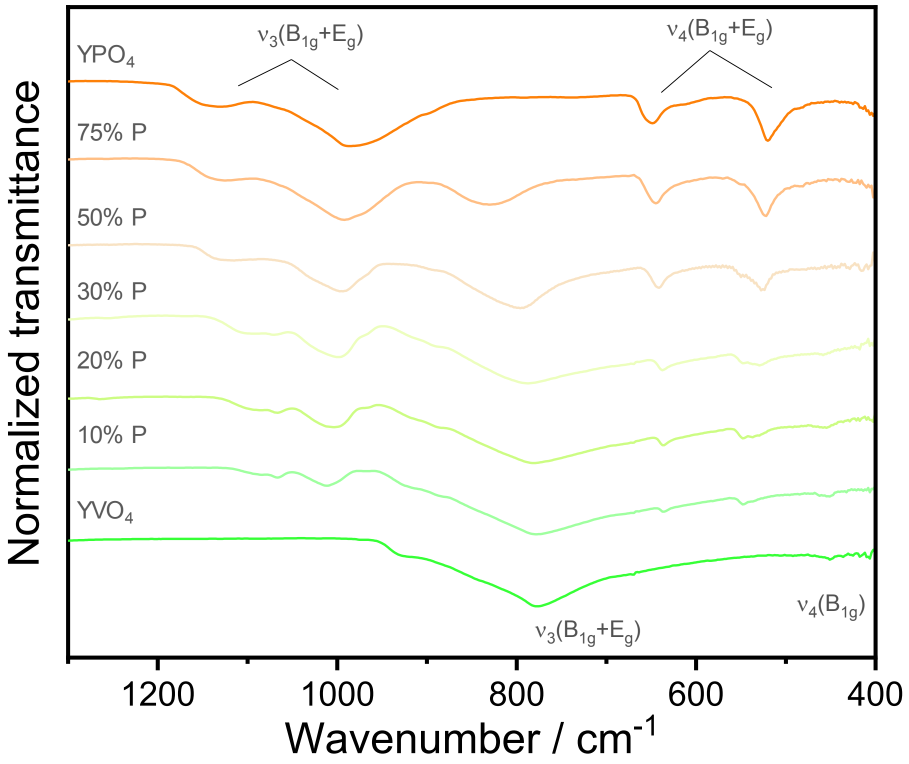

Figure S2. Infrared spectra (FTIR-ATR) of $Y_{0.999}Eu_{0.001}V_{1-x}P_xO_4$ nanocrystals (x = 0, 0.1, 0.2, 0.3, 0.5, 0.75, and 1) prepared by hydrothermal synthesis using Na_3VO_4 and $Na_3cit.2H_2O$.

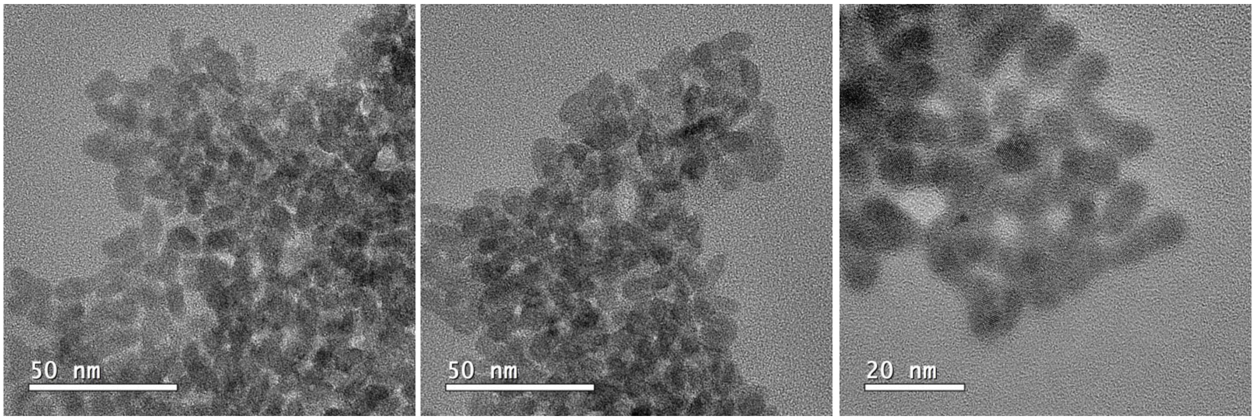

Figure S3. Transmission electron microscopy images of $Y(V_{0.8}P_{0.2})O_4$ nanocrystals prepared by hydrothermal synthesis using Na_3VO_4 and $Na_3cit.2H_2O$.

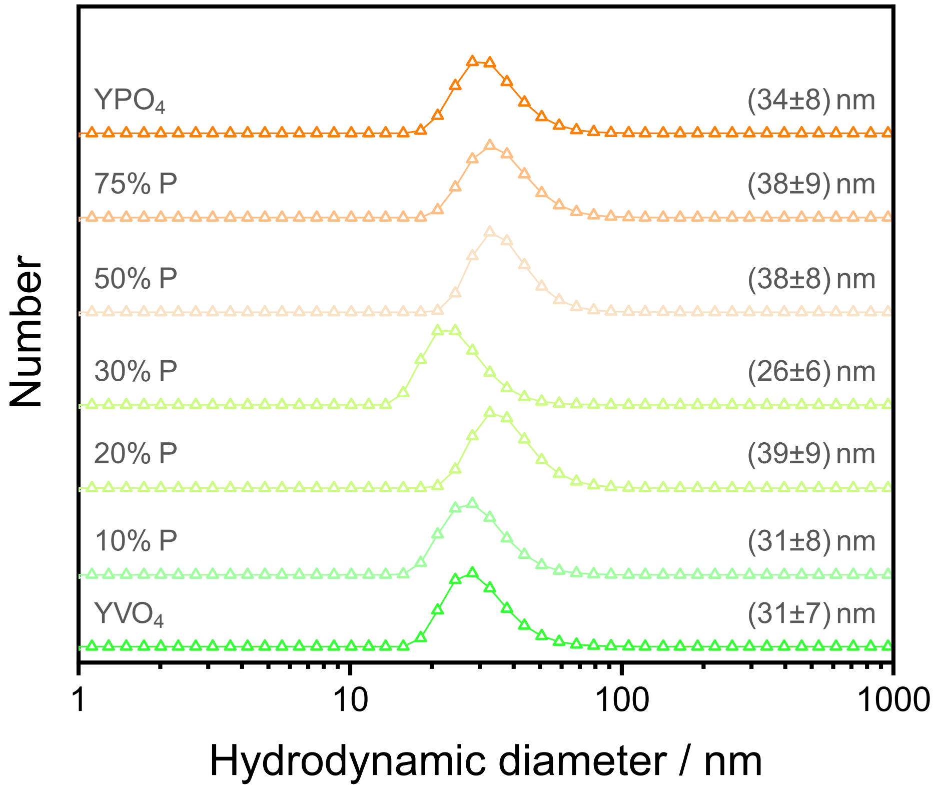

Figure S4. Dynamic light scattering (DLS) size distributions (number-weighted) of $Y_{0.999}Eu_{0.001}V_{1-x}P_xO_4$ nanocrystals prepared by hydrothermal synthesis using Na_3VO_4 and $Na_3cit.2H_2O$. Before analysis, particle suspensions (~0.5 mmol L^{-1} in PO_4^{3-}/VO_4^{3-} ions) were stabilized with poly(ammonium acrylate) aqueous solution (80 mmol L^{-1}, pH = 8.5 – 9). The V(P):PAA molar ratio was 1:0.05.

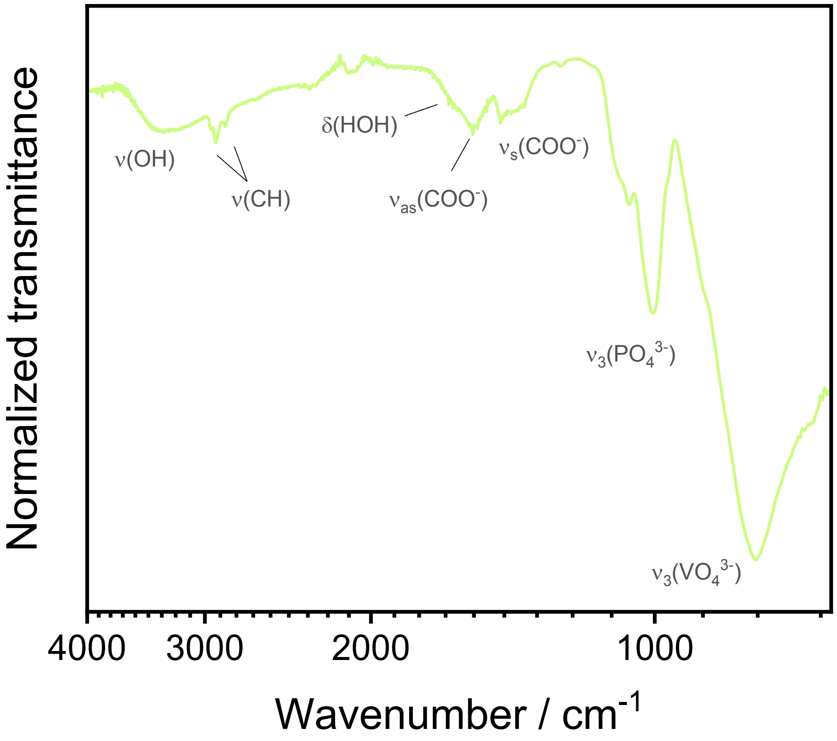

Figure S5. FTIR-ATR spectrum of $Y_{0.999}Eu_{0.001}(V_{0.8}P_{0.2})O_4$ nanocrystals showing the presence of citrate groups at the surface.

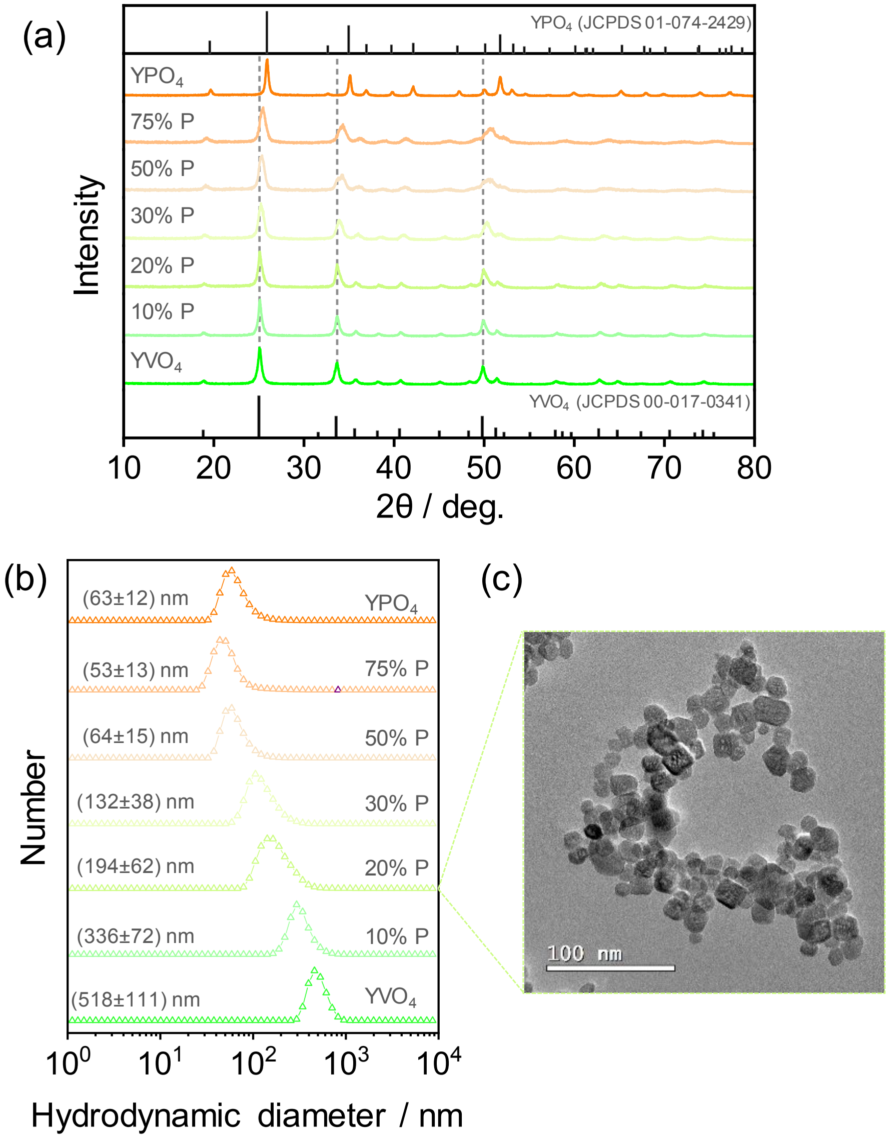

Figure S6. (a) Powder XRD diffractograms and (b) DLS size distributions (number-weighted) of $Y_{0.999}Eu_{0.001}(V_{1-x}P_x)O_4$ nanocrystals prepared by hydrothermal synthesis with $NaVO_3$. (c) TEM image of the $Y_{0.999}Eu_{0.001}(V_{0.8}P_{0.2})O_4$ nanoparticles.

3. THERMOMETRIC PROPERTIES

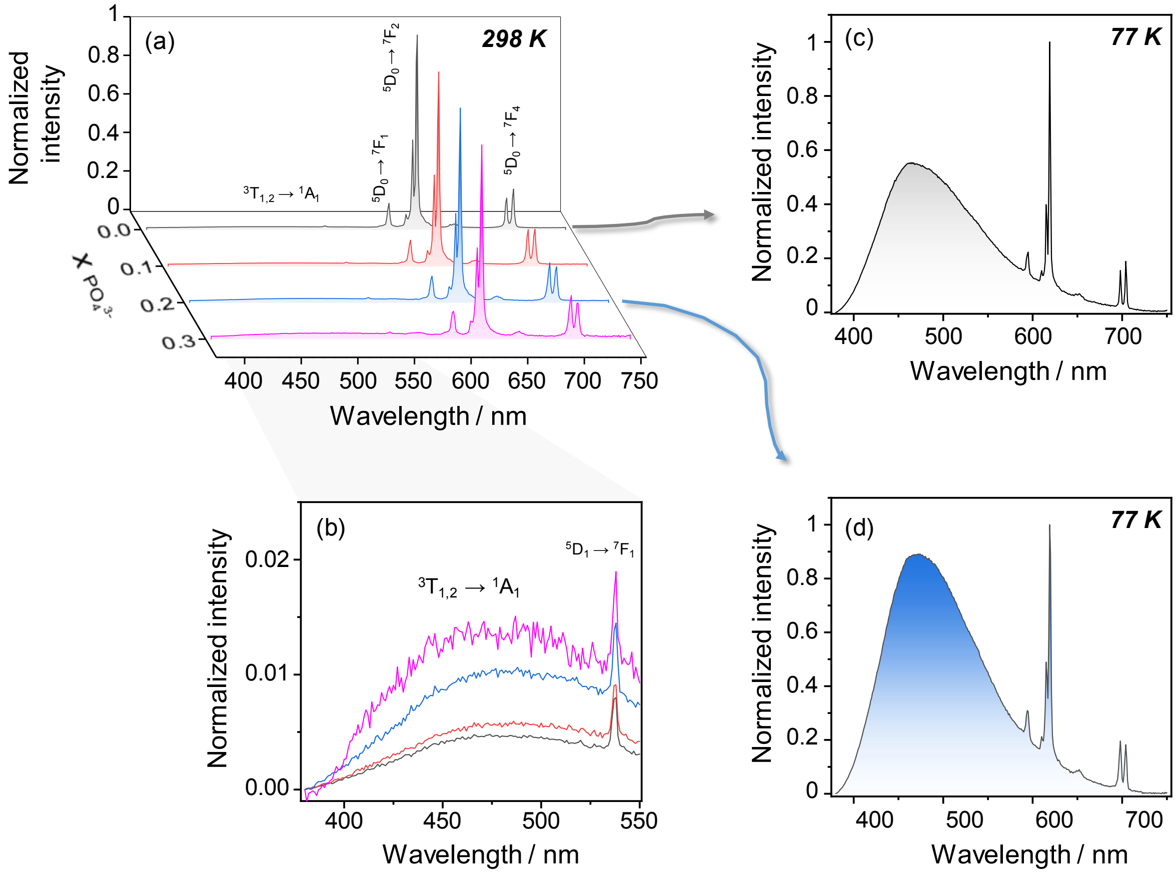

Figure S7. Luminescent properties of the $Y_{0.999}Eu_{0.001}(V_{1-x}P_x)O_4$ nanocrystals prepared by hydrothermal synthesis using Na_3VO_4 and $Na_3cit.2H_2O$. (a) Room-temperature emission spectra (λ_{exc} = 280 nm) showing the characteristics VO_4^{3-} ($^3T_{1,2} \rightarrow {}^1A_1$) and Eu^{3+} ($^5D_0 \rightarrow {}^7F_J$, J = 1–4) emissions. (b) Spectral amplification in the 380 – 550 nm region evidencing the VO_4^{3-} emission. Emission spectra of the (c) $Y_{0.999}Eu_{0.001}VO_4$ and (d) $Y_{0.999}Eu_{0.001}(V_{0.8}P_{0.2})O_4$ solids at 77 K.

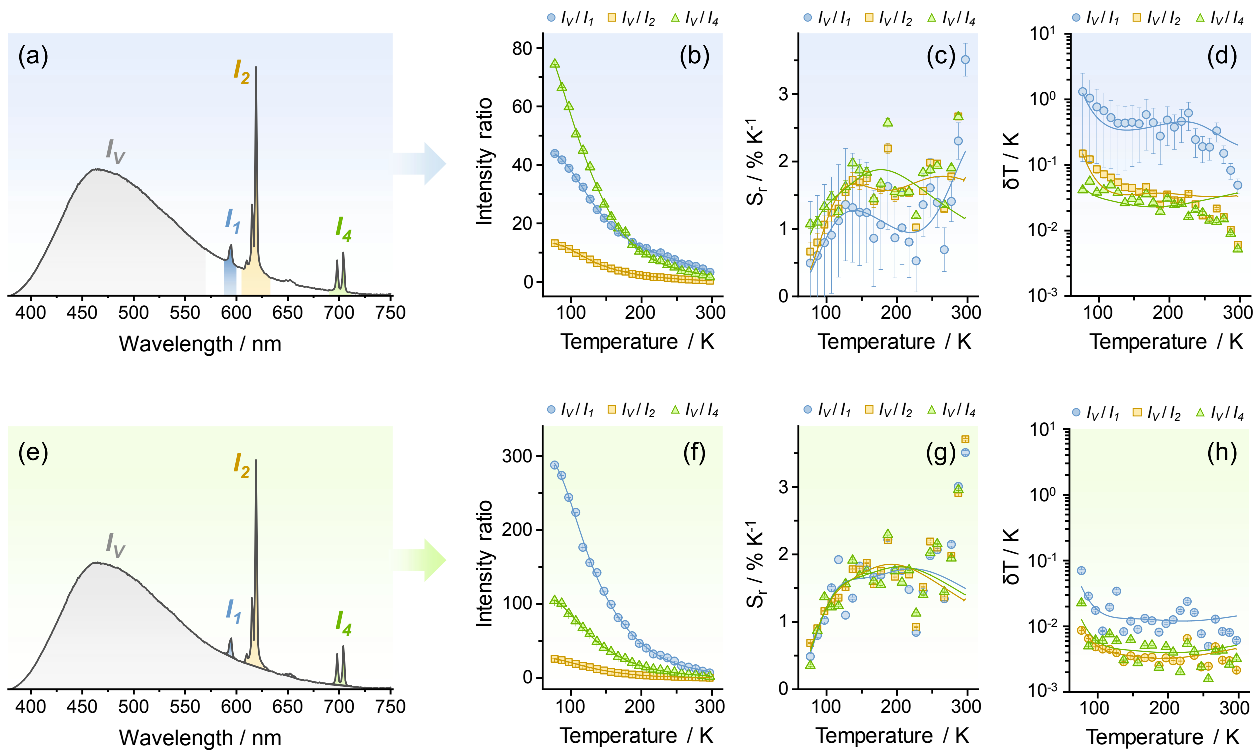

Figure S8. Impact of background emission on the thermometric performance of $Y_{0.999}Eu_{0.001}VO_4$ nanocrystals. (a),(e) Emission spectra (λ_{exc} = 280 nm, 77 K) illustrating the two spectral processing approaches to derive the intensity ratios. I_V denotes the integrated intensity of the $^3T_{1,2}\rightarrow{}^1A_1$ VO_4^{3-} transitions, whereas I_1, I_2, and I_4 correspond to the integrated intensities of the $^5D_0\rightarrow{}^7F_{1,2,4}$ Eu^{3+} transitions, respectively. Intensity ratios were calculated (a-d) neglecting signal superposition and (e-h) including a correction of the VO_4^{3-} emission background to compute the intensities of the Eu^{3+} signals. Temperature dependence of the (b),(f) intensity ratios, (c),(g) relative thermal sensitivities (S_r), and (d),(h) temperature uncertainties (δT). Solid lines in (b),(f) represent the best fits using Equation (1) (r^2 > 0.998), while solid lines in (c),(d),(g),(h) correspond to the mathematical derivation to model S_r and δT. Fitting parameters are summarized in Table S2.

Herein, errors for each intensity ratio were determined following the procedure described by Brites et al.[4] The relative uncertainty over the determination of Δ ($\delta\Delta/\Delta$) was calculated using Equation S1:

$$\frac{\delta\Delta}{\Delta} = \sqrt{\left(\frac{\delta I_V}{I_V}\right)^2 + \left(\frac{\delta I_J}{I_J}\right)^2} \quad \text{(S1)}$$

where $\delta I_V/I_V$ and $\delta I_J/I_J$ correspond to the signal-to-noise ratios (SNR) of the VO_4^{3-} and $^5D_0 \rightarrow {}^7F_J$ (J=1, 2 or 4) Eu^{3+} emissions, respectively. The $\delta I_V/I_V$ and $\delta I_J/I_J$ were obtained by dividing the readout fluctuations of the baseline (average of five points) by the maximum intensity value.

The errors in the determination of the relative thermal sensitivity (σ_{S_r}) and temperature uncertainty (σ_T) were accessed by Equation S2:

$$\frac{\sigma_{\delta T}}{\delta T} = \frac{\sigma_{S_r}}{\delta S_r} = \sqrt{2}\frac{\delta\Delta}{\Delta} \quad \text{(S2)}$$

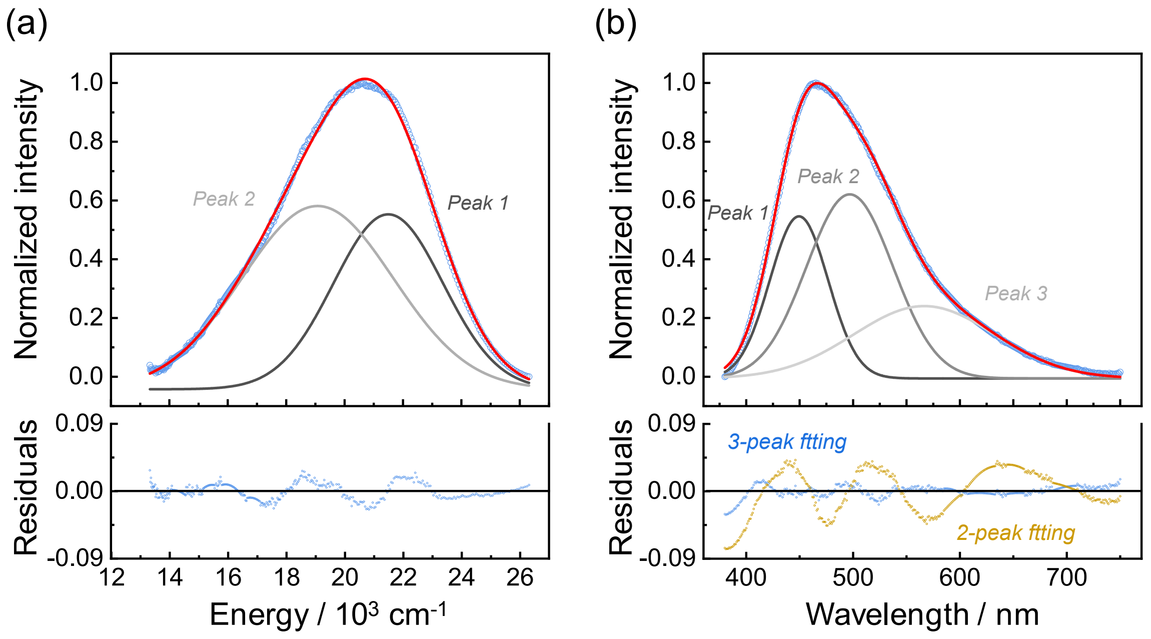

Figure S9. Emission spectrum (λ_{exc} = 280 nm, 77 K) of the $Y_{0.999}Eu_{0.001}(V_{0.8}P_{0.2})O_4$ nanoparticles in (a) energy and (b) wavelength units after removal of Eu^{3+} emissions. The resultant VO_4^{3-} emission was deconvoluted into two ($r^2 > 0.999$) and three ($r^2 > 0.999$) Gaussian components as a function of energy and wavelength, respectively. Red lines represent the fit envelope and the corresponding residuals are shown at the bottom part. The residuals originating from the deconvolution of the vanadate band in wavelength into two Gaussian components are also depicted at the bottom part of (b).

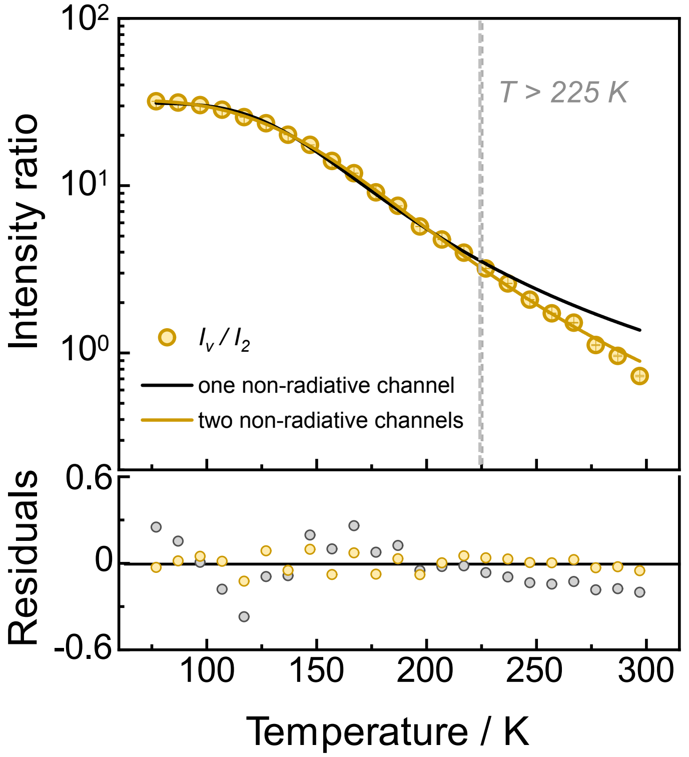

Figure S10. Temperature dependence of the intensity ratio involving the VO_4^{3-} and $^5D_0 \rightarrow ^7F_2$ Eu^{3+} emissions (I_V/I_2). Solid lines correspond to the best fits considering one (black line) and two (dark yellow line) non-radiative recombination channels in the Mott-Seitz model. For temperatures higher than 225 K, the experimental data were appropriately modeled by two thermal quenching pathways, as expected for the $^3T_{1,2} \rightarrow ^1A_1$ VO_4^{3-} transitions.

Table S2. Fitting parameters obtained from the thermal dependence of the Δ = I_V/I_1, I_V/I_2, and I_V/I_4 ratios for the $Y_{0.999}Eu_{0.001}VO_4:Eu^{3+}$ and $Y_{0.999}Eu_{0.001}(V_{0.8}P_{0.2})O_4$ nanocrystals: Δ parameter when T→0 K (Δ_0), ratio between the non-radiative and radiative rates for the $^3T_2 \rightarrow {}^1A_1$ (α_1) and $^3T_1 \rightarrow {}^1A_1$ (α_2) transitions, and activation energies for the non-radiative recombination channels of the corresponding transitions (ΔE_1 and ΔE_2). The values correspond to the experimental fits using Equation (1) after eliminating the interference of background emissions; r^2 denotes the correlation coefficient

	Δ_0	α_1	α_2	ΔE_1	ΔE_2	r^2
$Y_{0.999}Eu_{0.001}VO_4$						
I_V/I_1	308±8	7150±1015	55±19	1180±162	360±33	0.9980
I_V/I_2	28±1	2876±140	38±9	978±92	328±23	0.9996
I_V/I_4	113±3	4449±424	47±16	1075±77	347±31	0.9988
$Y_{0.999}Eu_{0.001}(V_{0.8}P_{0.2})O_4$						
I_V/I_1	321±5	12475±2426	140±45	1281±174	530±68	0.9981
I_V/I_2	32±1	4081±183	50±15	1019±88	438±44	0.9997
I_V/I_4	113±3	5834±718	63±27	1088±135	453±91	0.9981

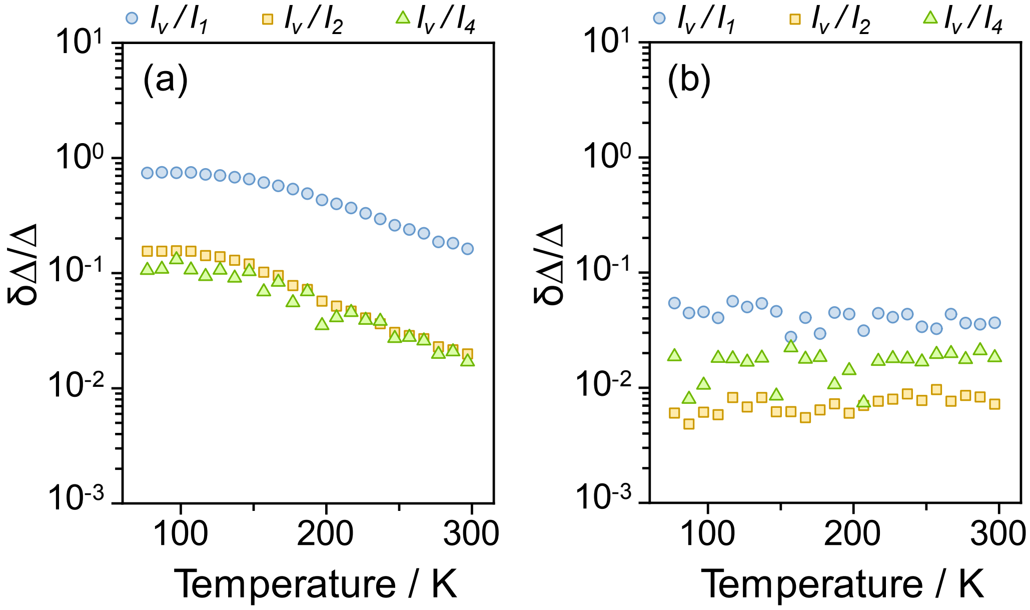

Figure S11. Temperature dependence of the relative uncertainty δΔ/Δ, where Δ = I_V/I_1, I_V/I_2, and I_V/I_4. Experimental points denote δΔ/Δ values for $Y(V_{0.8}P_{0.2})O_4:Eu^{3+}$ nanocrystals in the (a) absence and (b) presence of background correction to eliminate the interference of the VO_4^{3-} emission from the Eu^{3+} integration limits.

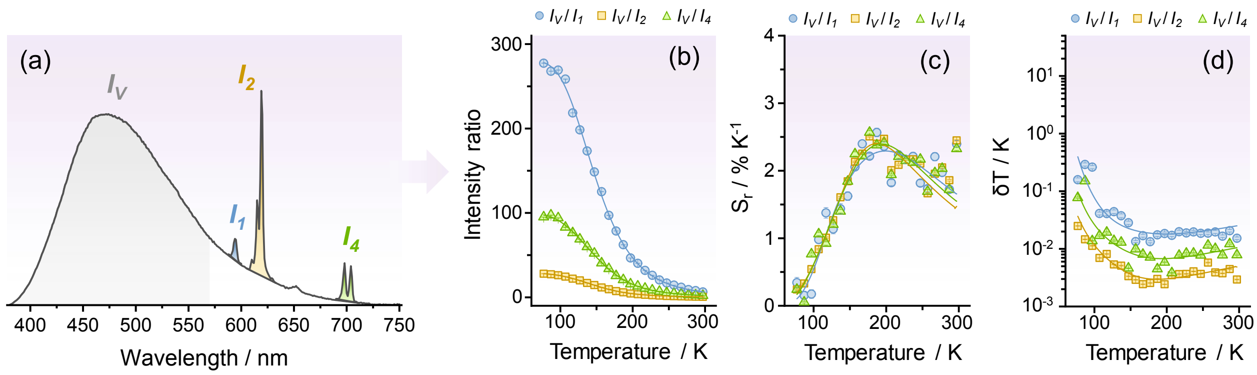

Figure S12. Alternative approach to eliminate the VO_4^{3-} emission contribution from the background. The only difference with respect to the treatment displayed in Figure 3e is the integration boundary of the VO_4^{3-} band (380 – 570 nm). (a) Emission spectrum (λ_{exc} = 280 nm) of the $Y_{0.999}Eu_{0.001}VO_4$ nanocrystals collected at 77 K. Temperature dependence of the (b) intensity ratios, (c) relative thermal sensitivities (S_r), and (d) temperature uncertainties (δT). This strategy enabled reliable thermometric correlations and afforded similar performances in comparison to the procedure involving the VO_4^{3-} emission envelope within 380 – 750 nm range.

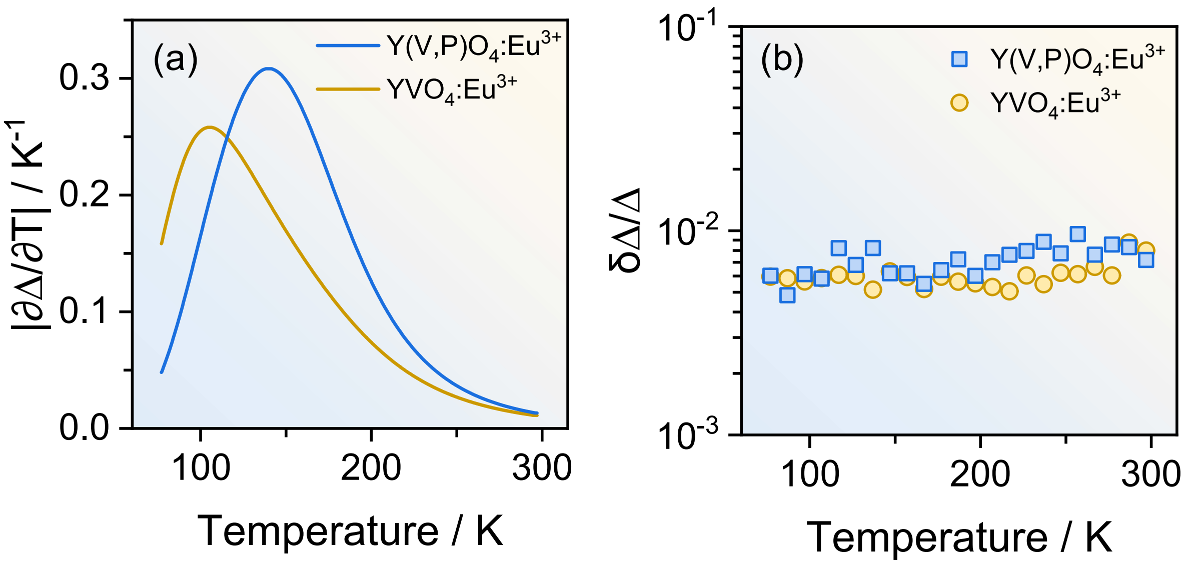

Figure S13. (a) Modulus of the first-order derivative of $\Delta = I_V/I_2$ parameter with respect to the temperature. (b) Relative uncertainty in the determination of $\Delta = I_V/I_2$ parameter ($\delta\Delta/\Delta$) for $Y_{0.999}Eu_{0.001}VO_4$ (circles) and $Y_{0.999}Eu_{0.001}(V_{0.8}P_{0.2})O_4$ (squares) nanocrystals.

Table S3. Comparative thermometric performance of illustrative examples of RE-based solid-state luminescent ratiometric thermometers: maximum relative sensitivities (S_m), temperatures for maximum relative sensitivities (T_m), measured temperature range (ΔT), operational ranges for relative thermal sensitivities higher than 1% K^{-1}, and maximum temperature uncertainty (δT_m)

Material	S_m / % K^{-1}	T_m / K	ΔT / K	ΔT for S_r >1% K^{-1} / K	δT_m / K	Ref.
$Y_{0.999}Eu_{0.001}(V_{0.8}P_{0.2})O_4$	2.27	191	77 – 297	127 – 287	0.55	This work
$Y_{0.999}Eu_{0.001}VO_4$	1.85	187	77 – 297	97 – 277	0.07	This work
$Gd_2(MoO_4)_3$:Eu^{3+}, Tb^{3+}	0.50	270	80 – 450	-	0.031	[5]
YVO_4:Yb^{3+},Nd^{3+}	0.24	163	123–423	-	-	[6]
$Ba_3(VO_4)_2$:Eu^{3+}	2.01	773	98 – 773	673 – 773	2.2	[7]
Sr_2GeO_4:Pr^{3+}	7.50	22	17 – 600	17 – 300	1.0	[8]
$La_2Zr_2O_7$:Pr^{3+}	0.41	165	85 – 705	-	> 4	[9]

4. IMPACT OF SIGNAL SUPERPOSITION ON THE THERMOMETRY QUALITY PARAMETERS

To prove that signal superposition within the integration limits underestimates the thermometric performance, we analyzed the emission profile of the $Y_{0.999}Eu_{0.01}(V_{0.8}P_{0.2})O_4$ sample at 77 K (Figure S14a). The integrated intensity ratios between the VO_4^{3-} emission (I_V) and each of the Eu^{3+} emissions ($I_J = I_1$, I_2, and I_4) depend on the correction of the VO_4^{3-} emission background. If correction is taken into account, the thermometric parameter, $\Delta_J(T)$, is given by:

$$\Delta_J(T) = \frac{I_V(T)}{I_J} \qquad (S3)$$

where I_J is less affected by temperature changes, as evidenced by Figure 2(c,d) in the main text. To simplify the following analysis, we consider I_J nearly temperature-independent. If correction is neglected, the $\Delta_J(T)$ parameter becomes an apparent thermometric parameter, $\Delta_J(T)^{AP}$, because:

$$\Delta_J(T)^{AP} = \frac{I_V(T)}{I_J + \varepsilon_J(T)} \qquad (S4)$$

where $\varepsilon_J(T)$ corresponds to a fraction of the VO_4^{3-} emission superposed to the $^5D_0 \rightarrow {}^7F_J$ Eu^{3+} transitions. Although $\varepsilon_J(T)$ and $\Delta_J(T)$ describe essentially the same thermal quenching of VO_4^{3-} emissions, each function presents a different mathematical meaning. The $\Delta_J(T)$ quantity defines an integrated intensity ratio between two emitting centers, one of which is less temperature dependent (I_J, Equation S5), while $\varepsilon_J(T)$ denotes a fraction of the absolute VO_4^{3-} emission as a function of temperature (Equations S6):

$$\Delta_J(T) = \frac{\Delta_0}{1 + \alpha_1 \exp\left(-\Delta E_1/k_B T\right) + \alpha_2 \exp\left(-\Delta E_2/k_B T\right)} \qquad (S5)$$

$$\varepsilon_J(T) = \frac{\Gamma_0{}^*}{1 + \alpha_1{}^* \exp\left(-\Delta E_1{}^*/k_B T\right) + \alpha_2{}^* \exp\left(-\Delta E_2{}^*/k_B T\right)} \tag{S6}$$

where $\Gamma_0{}^*$ is the integrated intensity when T → 0 K. This parameter varies regarding the choice of the integration boundaries for the $^5D_0 \rightarrow {}^7F_J$ (J=1, 2 or 4) Eu^{3+} emissions (Figure S14b).

Based on Equations S3 and S4, we can rearrange $\Delta_J(T)$ and $\Delta_J(T)^{AP}$ as follows:

$$\Delta_J(T)^{AP} = \Delta_J(T)\frac{I_J}{I_J + \varepsilon_J(T)} \tag{S7}$$

and adding Equation S6 in Equation S7, we can rewrite:

$$\Delta_J(T)^{AP} = \Delta_J(T)\frac{I_J}{I_J + \frac{\Gamma_0{}^*}{1 + \alpha_1{}^* \exp\left(-\Delta E_1{}^*/k_B T\right) + \alpha_2{}^* \exp\left(-\Delta E_2{}^*/k_B T\right)}} \tag{S8}$$

From Equation S7, we note that when $\varepsilon_J(T)$ tends to zero, $\Delta_J(T)^{AP}$ equals $\Delta_J(T)$, but if $\varepsilon_J(T) > 0$, then $\Delta_J(T)^{AP} < \Delta_J(T)$ (Figure 3b,f in the main text). Consequently, we should expect a higher relative thermal sensitivity (S_r) coming from the $\Delta_J(T)^{AP}$ parameter due to the inverse relationship between S_R and $\Delta(T)$ (Equation S9):

$$S_r(T) = \frac{1}{\Delta(T)}\left|\frac{d\Delta(T)}{dT}\right| \tag{S9}$$

However, the opposite trend is observed: the $\Delta_J(T)^{AP}$ parameter provides lower S_r values compared to those obtained from $\Delta_J(T)$ (Figure 3c,g in the main text). This demonstrates the dominant contribution of the $|d\Delta(T)/dT|$ term for the final thermal sensitivities. In other words, $S_r(T)^{AP} < S_r(T)$ because $|d\Delta_J(T)^{AP}/dT| < |d\Delta_J(T)/dT|$. To verify this statement, we calculated $|d\Delta_J(T)/dT|$ and $|d\Delta_J(T)^{AP}/dT|$ through the abovementioned Equations S5 and S8, resulting in the follow expressions:

$$\left|\frac{d\Delta_J(T)}{dT}\right| = \frac{\Delta_0\left(\frac{\Delta E_1}{k_B}\alpha_1 exp\left(-\frac{\Delta E_1}{k_B T}\right) + \frac{\Delta E_2}{k_B}\alpha_2 exp\left(-\frac{\Delta E_2}{k_B T}\right)\right)}{T^2\left(1 + \alpha_1 exp\left(-\frac{\Delta E_1}{k_B T}\right) + \alpha_2 exp\left(-\frac{\Delta E_2}{k_B T}\right)\right)^2} \qquad \text{(S10)}$$

and:

$$\left|\frac{d\Delta_J(T)^{AP}}{dT}\right| = \left[\frac{\Delta_0\left(\frac{\Delta E_1}{k_B}\alpha_1 exp\left(-\frac{\Delta E_1}{k_BT}\right)+\frac{\Delta E_2}{k_B}\alpha_2 exp\left(-\frac{\Delta E_2}{k_BT}\right)\right)}{T^2\left(1+\alpha_1 exp\left(-\frac{\Delta E_1}{k_BT}\right)+\alpha_2 exp\left(-\frac{\Delta E_2}{k_BT}\right)\right)^2}\frac{I_J\left(1+\alpha_1^* exp\left(-\frac{\Delta E_1^*}{k_BT}\right)+\alpha_2^* exp\left(-\frac{\Delta E_2^*}{k_BT}\right)\right)}{I_J\left(1+\alpha_1^* exp\left(-\frac{\Delta E_1^*}{k_BT}\right)+\alpha_2^* exp\left(-\frac{\Delta E_2^*}{k_BT}\right)\right)+\Gamma_0^*}\right]$$

$$-\left[\frac{\Delta_0\Gamma_0^* I_J\left(\frac{\Delta E_1^*}{k_B}\alpha_1^* exp\left(-\frac{\Delta E_1^*}{k_BT}\right)+\frac{\Delta E_2^*}{k_B}\alpha_2^* exp\left(-\frac{\Delta E_2^*}{k_BT}\right)\right)}{T^2\left(I_J\left(1+\alpha_1^* exp\left(-\frac{\Delta E_1^*}{k_BT}\right)+\alpha_2^* exp\left(-\frac{\Delta E_2^*}{k_BT}\right)\right)+\Gamma_0^*\right)^2\left(1+\alpha_1 exp\left(-\frac{\Delta E_1}{k_BT}\right)+\alpha_2 exp\left(-\frac{\Delta E_2}{k_BT}\right)\right)}\right] \quad (S11)$$

The first term of Equation S11 contains $|d\Delta_J(T)/dT|$, so substituting Equation S10 in the above expression:

$$\left|\frac{d\Delta_J(T)^{AP}}{dT}\right| = \left[\left|\frac{d\Delta_J(T)}{dT}\right|\frac{I_J\left(1+\alpha_1^* exp\left(-\frac{\Delta E_1^*}{k_BT}\right)+\alpha_2^* exp\left(-\frac{\Delta E_2^*}{k_BT}\right)\right)}{I_J\left(1+\alpha_1^* exp\left(-\frac{\Delta E_1^*}{k_BT}\right)+\alpha_2^* exp\left(-\frac{\Delta E_2^*}{k_BT}\right)\right)+\Gamma_0^*}\right]$$

$$-\left[\frac{\Delta_0\Gamma_0^* I_J\left(\frac{\Delta E_1^*}{k_B}\alpha_1^* exp\left(-\frac{\Delta E_1^*}{k_BT}\right)+\frac{\Delta E_2^*}{k_B}\alpha_2^* exp\left(-\frac{\Delta E_2^*}{k_BT}\right)\right)}{T^2\left(I_J\left(1+\alpha_1^* exp\left(-\frac{\Delta E_1^*}{k_BT}\right)+\alpha_2^* exp\left(-\frac{\Delta E_2^*}{k_BT}\right)\right)+\Gamma_0^*\right)^2\left(1+\alpha_1 exp\left(-\frac{\Delta E_1}{k_BT}\right)+\alpha_2 exp\left(-\frac{\Delta E_2}{k_BT}\right)\right)}\right] \quad (S12)$$

We can neglect the second term on the right side of Equation S12 because it is lower than 1 for all considered temperatures. Thus, we finally achieve Equation S13:

$$\left|\frac{d\Delta_J(T)^{AP}}{dT}\right| = \left|\frac{d\Delta_J(T)}{dT}\right| \frac{I_J\left(1+\alpha_1^* exp\left(-\frac{\Delta E_1^*}{k_B T}\right)+\alpha_2^* exp\left(-\frac{\Delta E_2^*}{k_B T}\right)\right)}{I_J\left(1+\alpha_1^* exp\left(-\frac{\Delta E_1^*}{k_B T}\right)+\alpha_2^* exp\left(-\frac{\Delta E_2^*}{k_B T}\right)\right)+\Gamma_0^*} \tag{S13}$$

The second term on the right side of this equation is lower than 1, as Γ_0* holds a positive value. Thus, $|d\Delta_J{}^{AP}/dT|$ is lower than $|d\Delta_J/dT|$ for all temperatures, resulting in an underestimation of the thermometry quality parameters (S_r and consequently δT).

We also note that this condition vanishes if the contribution of the VO_4^{3-} band to the integration limits of the Eu^{3+} signals diminishes. To better illustrate this behavior, we calculated the ε_J(T)/I_J ratio, which indicates the degree of signal superposition embedded in each intensity ratio (*i.e.*, I_V/I_1, I_V/I_2, and I_V/I_4). The progressive decrease from the ε_1/I_1 ratio toward the ε_4/I_4 ratio (Figure S14c) aligns with the decreased superposition between the VO_4^{3-} emission and the $^5D_0\rightarrow{}^7F_4$ Eu^{3+} emission. As a result, the I_v/I_4 ratio yields a $|d\Delta_J{}^{AP}/dT|$ that essentially equals $|d\Delta_J/dT|$ (Figure S14d). This adjustment enables the accuracy of the thermometric parameters derived from this ratio, as discussed in the main text.

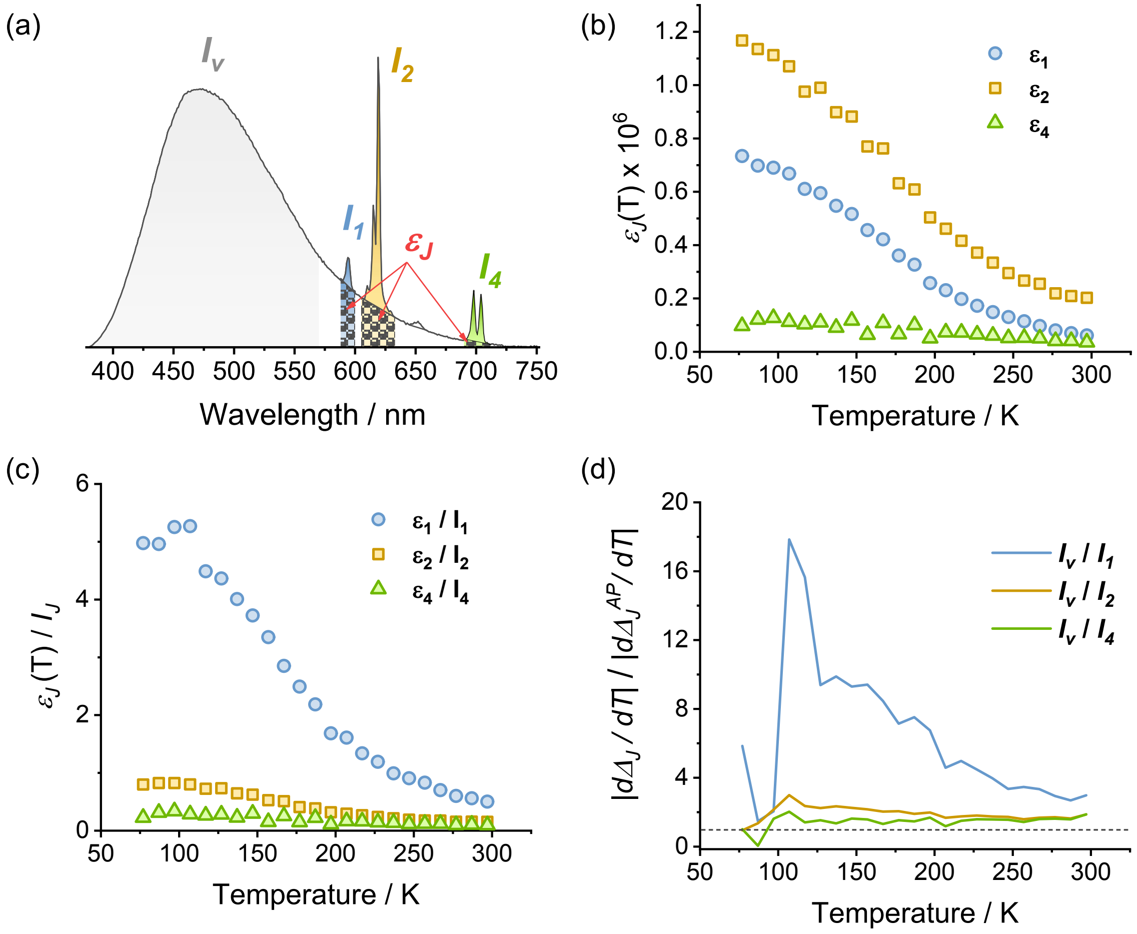

Figure S14. (a) Emission spectrum (λ_{exc} = 280 nm, 77 K) of $Y_{0.999}Eu_{0.001}(V_{0.80}P_{0.20})O_4$ nanocrystals showing the VO_4^{3-} (I_v) and Eu^{3+} (I_1: $^5D_0 \rightarrow {}^7F_1$, I_2: $^5D_0 \rightarrow {}^7F_2$, and I_4: $^5D_0 \rightarrow {}^7F_4$) emissions with the corresponding superposed signals, ε_J(T). Dependence of (b) ε_J(T) parameter and (c) ε_J(T)/ I_J (J=1, 2, and 4) ratio against temperature. (d) Ratio between the derivatives of the real ($|d\Delta_J(T)/dT|$) and apparent ($|d\Delta_J(\mathrm{T})^{AP}/dT|$) thermometric parameter. Horizontal dashed line corresponds to the case where $|d\Delta_J(\mathrm{T})^{AP}/dT| = |d\Delta_J(T)/dT|$.

REFERENCES

(1) Ray, S.; Nair, G. B.; Tadge, P.; Malvia, N.; Rajput, V.; Chopra, V.; Dhoble, S. J. Size and Shape-Tailored Hydrothermal Synthesis and Characterization of Nanocrystalline $LaPO_4:Eu^{3+}$ Phosphor. *J. Lumin.* **2018**, *194*, 64–71.

(2) Xu, Z.; Kang, X.; Li, C.; Hou, Z.; Zhang, C.; Yang, D.; Li, G.; Lin, J. Ln^{3+} (Ln = Eu, Dy, Sm, and Er) Ion-Doped YVO_4 Nano/Microcrystals with Multiform Morphologies: Hydrothermal Synthesis, Growing Mechanism, and Luminescent Properties. *Inorg. Chem.* **2010**, *49*, 6706–6715.

(3) Lutterotti, L.; Matthies, S.; Wenk, H.-R. MAUD: A Friendly Java Program for Material Analysis Using Diffraction. *IUCr Newsl. CPD* **1999**, *21*, 14–15.

(4) Brites, C. D. S.; Millán, A.; Carlos, L. D. Lanthanides in Luminescent Thermometry. In *Handbook on the Physics and Chemistry of Rare Earths*; Bünzli, J.-C. G., Pecharsky, V. K., Eds.; Vol. 49; Elsevier: Amsterdam, 2016; pp 339–427.

(5) Han, L.; Liu, J.; Liu, P.; Li, B.; Li, X.; Xu, Y. Dual-Emissive Eu^{3+}, Tb^{3+} Co-Doped $Gd_2(MoO_4)_3$ Phosphor for Optical Thermometry Application. *J. Phys. Chem. Solids* **2021**, *153*, 110032.

(6) Marciniak, L.; Bednarkiewicz, A.; Trejgis, K.; Maciejewska, K.; Elzbieciak, K.; Ledwa, K. Enhancing the Sensitivity of a Nd^{3+},Yb^{3+}:YVO_4 Nanocrystalline Luminescent Thermometer by Host Sensitization. *Phys. Chem. Chem. Phys.* **2019**, *21*, 10532–10539.

(7) Kolesnikov, I. E.; Mamonova, D. V.; Kurochkin, M. A.; Khodasevich, M. A.; Medvedev, V. A.; Kolesnikov, E. Y.; Manshina, A. A. Single vs. Mutliparametric Luminescence Thermometry: The Case of Eu^{3+}-Doped $Ba_3(VO_4)_2$ Nanophosphors. *J. Mater. Chem. C* **2023**, *11*, 14814–14825.

(8) Brites, C. D. S.; Fiaczyk, K.; Ramalho, J. F. C. B.; Sójka, M.; Carlos, L. D.; Zych, E.

Widening the Temperature Range of Luminescent Thermometers through the Intra- and Interconfigurational Transitions of Pr^{3+}. *Adv. Opt. Mater.* **2018**, *6*, 1701318.

(9) Jahanbazi, F.; Wang, Y.; Dorman, J. A.; Mao, Y. $La_2Zr_2O_7$:Pr^{3+} Nanoparticles for Luminescence Thermometry Based on a Single Parameter over a Wide Temperature Range of 620 K. *J. Alloys Compd.* **2022**, *911*, 165013.
